# A proteome-wide protein interaction map for *Campylobacter jejuni*

**DOI:** 10.1186/gb-2007-8-7-r130

**Published:** 2007-07-05

**Authors:** Jodi R Parrish, Jingkai Yu, Guozhen Liu, Julie A Hines, Jason E Chan, Bernie A Mangiola, Huamei Zhang, Svetlana Pacifico, Farshad Fotouhi, Victor J DiRita, Trey Ideker, Phillip Andrews, Russell L Finley

**Affiliations:** 1 Center for Molecular Medicine and Genetics, Wayne State University School of Medicine, Detroit, MI, USA 48201; 2 Department of Biochemistry and Molecular Biology, Wayne State University School of Medicine, Detroit, MI, USA 48201; 3 Department of Bioengineering and Program in Bioinformatics, University of California at San Diego, San Diego, CA, USA 92093; 4 Department of Computer Science, Wayne State University, Detroit, MI, USA 48201; 5 Department of Microbiology and Immunology, University of Michigan Medical School, Ann Arbor, MI, USA 48109; 6 Department of Biological Chemistry, University of Michigan Medical School, Ann Arbor, MI, USA 48109

## Abstract

**Background:**

Data from large-scale protein interaction screens for humans and model eukaryotes have been invaluable for developing systems-level models of biological processes. Despite this value, only a limited amount of interaction data is available for prokaryotes. Here we report the systematic identification of protein interactions for the bacterium *Campylobacter jejuni*, a food-borne pathogen and a major cause of gastroenteritis worldwide.

**Results:**

Using high-throughput yeast two-hybrid screens we detected and reproduced 11,687 interactions. The resulting interaction map includes 80% of the predicted *C. jejuni *NCTC11168 proteins and places a large number of poorly characterized proteins into networks that provide initial clues about their functions. We used the map to identify a number of conserved subnetworks by comparison to protein networks from *Escherichia coli *and *Saccharomyces cerevisiae*. We also demonstrate the value of the interactome data for mapping biological pathways by identifying the *C. jejuni *chemotaxis pathway. Finally, the interaction map also includes a large subnetwork of putative essential genes that may be used to identify potential new antimicrobial drug targets for *C. jejuni *and related organisms.

**Conclusion:**

The *C. jejuni *protein interaction map is one of the most comprehensive yet determined for a free-living organism and nearly doubles the binary interactions available for the prokaryotic kingdom. This high level of coverage facilitates pathway mapping and function prediction for a large number of *C. jejuni *proteins as well as orthologous proteins from other organisms. The broad coverage also facilitates cross-species comparisons for the identification of evolutionarily conserved subnetworks of protein interactions.

## Abstract 2

'Systematic identification of protein interactions for the bacterium *Campylobacter jejuni *using high-throughput yeast two-hybrid screens detected interactions for 80% of the organism's proteins.

## Background

A catalog of all the protein interactions that occur in an organism could provide a useful starting point for understanding the functions of proteins and entire biological systems. Several research groups have performed large-scale screens with the goal of identifying all of the protein interactions, or the interactome, for a given organism. One productive approach has been to co-affinity purify (co-AP) members of protein complexes using affinity-tagged bait proteins and then to identify the complex members using mass spectrometry (MS). This approach has been particularly useful for single-cell model organisms like *Escherichia coli *and *Saccharomyces cerevisiae*, in which large sets of affinity-tagged proteins can be expressed readily and co-AP/MS can be performed on large quantities of cells [1-6]. A complementary approach that detects binary protein interactions rather than protein complexes is the yeast two-hybrid system [7]. In contrast to the co-AP/MS studies, large-scale yeast two-hybrid screens measure interactions in an artificial setting, the yeast nucleus, with the goal of mapping all of the possible specific binary interactions that may occur *in vivo*. Large-scale yeast two-hybrid screens have been used to probe the interactomes of a wide range of organisms from viruses to humans (see [8-10] for reviews). The yeast two-hybrid screens and the co-AP/MS studies provide at least a static picture of protein interactions that may occur under one or a defined set of *in vivo *conditions. The resulting interaction maps can provide a framework for understanding pathways and molecular machines, particularly when combined with other types of functional genomics data, including gene phenotypes and dynamic information such as gene expression, protein expression, and protein localization data.

Very few bacterial species have been analyzed at the proteome level for protein interactions. For example, large-scale systematic determination of binary protein interactions has been described for only one bacterium to date, *Helicobacter pylori *[11]. That study resulted in interactions covering 46% of the *H. pylori *proteome (Additional data file 1). Meanwhile, *E. coli *is the only bacterium for which protein complex purifications have been applied at the proteome scale [1,6]. Binary protein interactions predicted from these studies include 80% of the *E. coli *proteome. With the immense number and diversity of different bacterial species that exist, a huge reservoir of prokaryotic protein interactions have yet to be sampled.

*Campylobacter jejuni *is a Gram-negative food-borne pathogen that is a major cause of gastroenteritis in humans [12]. Infection with *C. jejuni *has also been associated with the autoimmune peripheral neuropathy known as Guillain Barré syndrome and immunoproliferative small intestinal disease [13-15]. Despite the importance of *C. jejuni *as a pathogen, much remains to be learned about its biology and mechanisms for causing disease. The functions of over 50% of the 1,654 proteins predicted to be encoded by the *C. jejuni *NCTC11168 genome are either unknown or poorly characterized, as implied by their unnamed gene status [16]. Clues about the functions of these proteins could come from protein interaction data. Most of the protein interaction data for *C. jejuni *come from small-scale experiments with individual proteins or from the somewhat less reliable method of predicting interactions based on measurements with orthologous proteins in other organisms. Despite the proven utility of protein interaction data, most of the *C. jejuni *proteins are not yet known or predicted to be involved in an interaction. Thus, interactome data could significantly aid *C. jejuni *research. Because co-AP/MS studies would be difficult for this organism we set out to map interactions using the two-hybrid system.

Here we report the results of a proteome-scale systematic screen of *C. jejuni *protein interactions. Using a comprehensive yeast two-hybrid approach we tested over 89% of the predicted *C. jejuni *NCTC11168 proteins for interactions and identified thousands of novel protein interactions covering 80% of the proteome. For each interaction we generated a confidence score that reflects its probability of being biologically relevant, resulting in 2,884 interactions with high confidence scores. We demonstrate how these data can be used to map pathways, generate hypotheses about protein function and network evolution, and to identify potential new drug targets. We have assembled all of the interactions from this study into a single comprehensive *C. jejuni *protein interaction database [17] that also contains computational predictions [18] and interolog [19] predictions based on *E. coli *and *H. pylori *protein interactions. The interaction data can be readily accessed and downloaded using the web-based application tool called IM Browser [20].

## Results

### Systematic identification of protein interactions for *C. jejuni *NCTC11168

**Figure 1:**
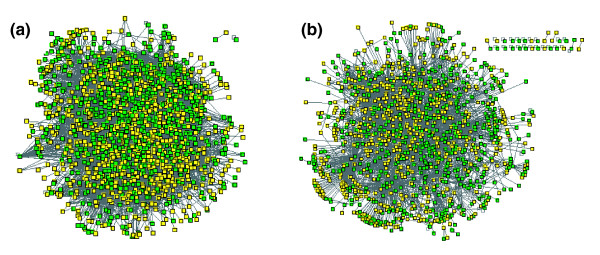
*C. jejuni *protein interaction networks. **(a) **The *C. jejuni *interaction dataset (CampyYTH v3.1), and **(b) **the higher confidence subset. In each case most of the proteins (square nodes) are connected into a single large network; the unconnected interactions are in the upper right of each panel. The networks in (a, b) connect over 79% (663 total) and 65% (548 total) of the unnamed and presumed poorly characterized proteins (yellow nodes), respectively.

We used a yeast two-hybrid pooled matrix approach [21,22] to screen for binary interactions among the predicted *C. jejuni *NCTC11168 proteins. We generated two arrays of yeast strains that express full length *C. jejuni *open reading frames (ORFs) fused to the LexA DNA-binding domain (BD) or a transcription activation domain (AD), respectively (Materials and methods). Over 89% of the predicted *C. jejuni *ORFs are represented in the arrays (Table 1). To sample all possible binary interactions, each member of the BD array was mated with pools containing approximately 96 AD strains and the resulting diploids were assayed for reporter activity. Each BD strain was then tested with every AD strain comprising the pools with which it was positive to identify the specific interacting protein pair. The activities of the two yeast two-hybrid reporters were independently quantified based on growth on selective media and color on X-Gal plates, as previously described [21]. Our screen initially detected a total of 16,022 putative interactions with above-threshold reporter scores (after subtraction of background activity for BD fusions capable of activating the reporters on their own). An additional 82 unique interactions were identified using a library screen (Materials and methods). We retested the combined 16,104 initial positives in individual one-on-one mating assays of BD strains and AD strains, and reproduced 11,687 of them. The majority of non-repeating interactions initially had shown low levels of reporter activity. The 11,687 repeated interactions were included in our final dataset (CampyYTH v3.1; Figure 1a).

**Table 1:** Summary of array generation and interaction testing

**ORFs total**	1,654
**ORFs cloned**	1,477
BD fusions	1,398
AD fusions	1,442
**Assays performed**	~336,000
Interactions*	11,687
BD proteins	637
AD proteins	1,248
Unique ORFs	1,321
Higher confidence interactions*^†^	2,884
BD proteins	589
AD proteins	923
Unique ORFs	1,067

*Interactions that repeated upon retesting. ^†^An additional 325 interactions received high confidence scores (> 0.5), but were not repeated in a second two-hybrid assay.

The interaction map includes all of the major protein types and is not significantly enriched for any particular gene classification (Additional data file 2). As expected, however, integral membrane proteins are slightly depleted (Additional data file 3), which was likely due to failure to reach the nucleus or improper folding in the nuclear environment. The high coverage (80% of the predicted proteome) can be attributed in part to the number of proteins tested, to the systematic pooled matrix approach, and to the use of regulated promoters to detect interactions with toxic proteins or proteins that activated the reporters on their own. For example, proteins toxic or inhibitory to yeast were successfully assayed by expressing the fusion proteins with an inducible rather than constitutive promoter [23]. Constitutive expression of inhibitory proteins can result in down regulation of the fusion proteins and loss of the ability to detect interactions [21]. In this study we found that 114 (7%) of the proteins in our array were either toxic or inhibitory to yeast (Additional data file 4). Nevertheless, we were able to detect over 700 interactions that involved these proteins, including the well-known GroES-GroEL interaction.

### Data quality and confidence scores

**Figure 2:**
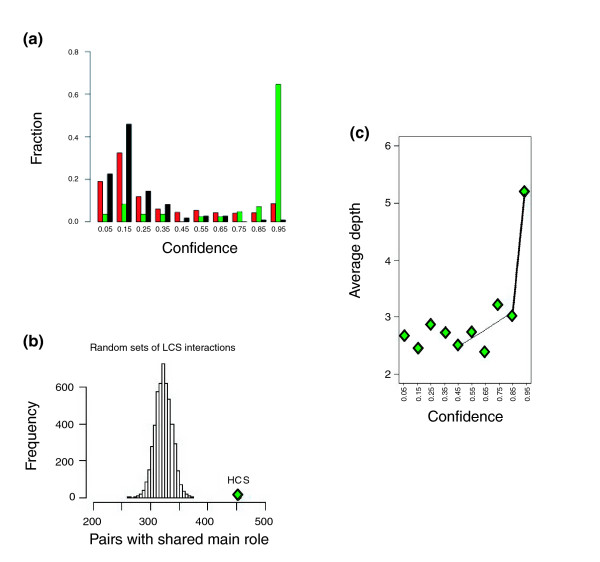
Confidence scores assigned to the *C. jejuni *protein interactions. **(a) **The distribution of confidence scores generated for the CampyYTH v3.1 protein interactions are shown in red. The distributions of scores for the training sets containing likely true positives (green) or true negatives (black) are also shown. **(b) **Protein interaction pairs with high confidence scores (HCS; confidence scores > 0.5) share the same functions significantly more frequently (*p *value < 3 × 10^-57^) than protein pairs comprising interactions with low confidence scores (LCS; confidence scores ≤ 0.5). Protein 'self' interactions were excluded from the analysis. **(c) **The average depth of shared GO biological process annotation was determined for the interactions comprising each confidence score bin. Higher confidence interactions generally involve proteins with the same functional annotation at greater depths of precision. The two dotted line segments are linearly fitted lines between average GO depth and bin number in two regions, from 0.5 to 0.9 and 0.9 to 1.0. Protein 'self' interactions were excluded from the analysis.

To help distinguish true positives from false positives we applied a statistical method to generate confidence scores for each interaction [24,25]. We used logistic regression to assign weights to a set of experimental interaction attributes based on how well they correlated with biological significance. Sets of putative true positives and false positives were used to train the scoring system on biological significance (Materials and methods). One interaction attribute that strongly correlated with putative true positives, for example, was the level of reporter activity, an attribute not determined in most previous large-scale two-hybrid screens. An attribute that correlated with false positives was the number of interactions per protein. The weighted attributes were combined in a model that assigned probability scores between 0 and 1 to each interaction. Choosing 0.5 as the threshold between low and high confidence interactions resulted in 2,884 (25%) of the reproduced interactions falling into the higher confidence set (Figure 2a), which covered 67% of the *C. jejuni *proteins. As an independent test of the confidence-scoring system, we demonstrated that interactions with higher confidence scores were significantly more likely to involve pairs of proteins known to function in the same biological process, as would be expected for true positives, than do an equal number of randomly selected low scoring interactions (p < 3 × 10^-57^; Figure 2b). For this analysis we used the biological role classifications that had been assigned previously [26], but which played no part in generating the confidence scores. Similarly, we found that the higher confidence interactions generally included more pairs of proteins that share more detailed Gene Ontology (GO) [27] functional annotations (Figure 2c). Combined, these analyses indicate that the confidence scores are a useful measure of biological significance to guide future studies.

**Figure 3:**
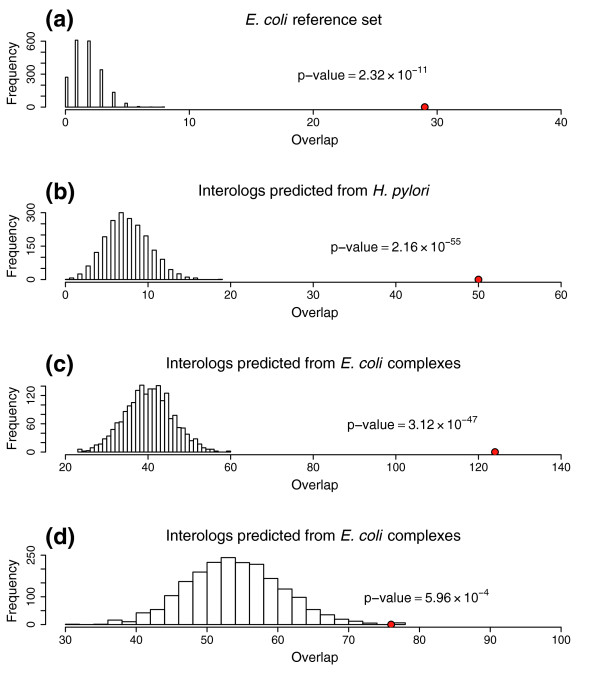
Comparison of the *C. jejuni *interaction map with other datasets. The interactions found in common, or overlap (red dots) between the *C. jejuni *two-hybrid map and interologs predicted from other organisms, were determined. This was compared to the overlap between the interolog datasets and 2,000 random maps generated by randomly switching pairs of links in the original yeast two-hybrid map, which preserves network degree distribution. **(a) **The two-hybrid map shared 28 interactions with a reference set containing 147 interologs of *E. coli *low-throughput literature-cited protein interactions, significantly greater than the overlap with the random maps. **(b) **There were 50 *C. jejuni *interactions shared with 1,165 interologs predicted from the *H. pylori *protein interaction dataset [11]. **(c) **There were 124 interactions shared with a set of 3,743 interologs predicted from a large-scale *E. coli *protein complex study [1]. **(d) **There were 76 interactions shared with a set of 4,056 interologs predicted from a second *E. coli *protein complex pull-down study [6]. A complete list of the predicted interologs used for these analyses can be found in Additional data file 12.

To further assess the quality of the *C. jejuni *interaction data we compared them to *E. coli *and *H. pylori *datasets presumed to be enriched for true positives. First, we considered a set of high-confidence *E. coli *protein interactions from literature-cited low-throughput experiments compiled within the Database of Interacting Proteins (DIP) [28]. Reciprocal best-match *C. jejuni *orthologs of the *E. coli *proteins were used to predict 147 conserved *C. jejuni *interactions or interologs. The overlap between the two-hybrid data and the predictions from the *E. coli *reference set was 28 of 147, significantly (*p *= 2 × 10^-11^) more than the overlap between the reference set and random maps with the same size and topology as the two-hybrid map (Figure 3a). The overlaps between our data and interologs predicted from *H. pylori *yeast two-hybrid data [11] or *E. coli *protein complexes [1,6] were also significantly greater than expected by chance (Figure 3b-d). Moreover, the fraction of *C. jejuni *data that overlaps with the reference set is similar to that for the *E. coli *and *H. pylori *high throughput datasets (Table 2). This analysis suggests that the *C. jejuni *yeast two-hybrid map has rates of true positives, false positives, and false negatives similar to the previous maps for *E. coli *and *H. pylori*.

**Table 2:** Comparison of *C. jejuni*, *H. pylori*, and *E. coli *protein interaction sets to an *E. coli *reference set containing 599 low-throughput literature-cited interactions*

	Reference set interologs^†^	Overlap with reference set interologs	Overlap (%) with total interactions detected for each study	Fraction of proteins in each study with orthologs in the reference set
*C. jejuni*	147	28 (19%)	0.24	7.9%
*C. jejuni *(HC)	147	27 (18%)	0.95	8.5%
*H. pylori*^‡^	84	10 (12%)	0.70	7.9%
*E. coli*^§^	599	81 (14%)	1.32	11.2%
*E. coli*^¶^	599	49 (8%)	0.44	11.2%

*The reference set was derived from DIP [28]. ^†^Interolog lists were generated separately for each organism based on BLASTP reciprocal best match determination of orthologs. ^‡^*H. pylori *interactions were detected by Rain *et al*. [11]. ^§^*E. coli *interactions were predicted from Butland *et al*. [1]. ^¶^*E. coli *interactions were predicted from Arrifuzzaman *et al*. [6]. HC, high confidence set.

### The *C. jejuni *protein interaction network

**Figure 4:**
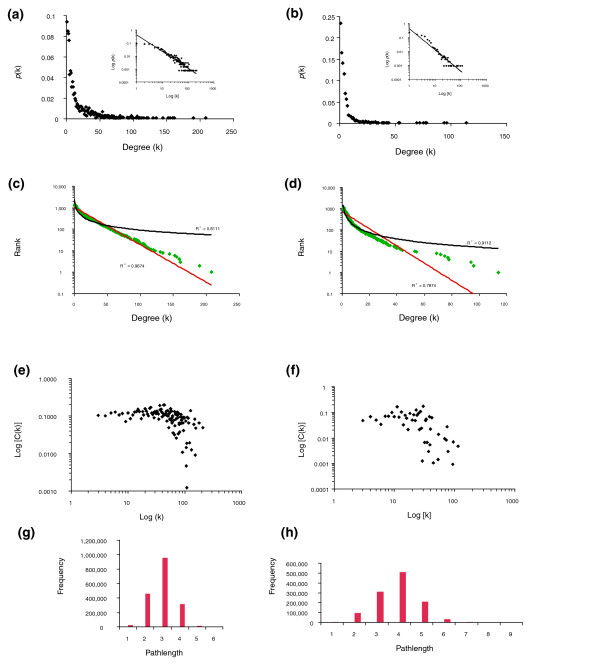
Characteristics of the *C. jejuni *protein network. **(a) **Degree frequency distribution for the entire two-hybrid dataset (CampyYTH v3.1). k = degree, the number of connections to a protein. P(k) = the probability that a node has k connections. A power law fit yields: y = 0.4153 x^-1.29^; R^2 ^= 0.88. **(b) **Degree frequency distribution for the high confidence dataset (confidence scores > 0.5). A power law fit yields: y = 482.2 x^-1.53^, R^2 ^= 0.89. **(c) **Rank-degree distribution for the entire two-hybrid dataset. The semi-log plot more closely fits an exponential curve (red line, R^2 ^= 0.97) than a power law curve (black line, R^2 ^= 0.81). **(d) **Rank-degree distribution for the high confidence data. The semi-log plot more closely fits a power law curve (black line, R^2 ^= 0.91) consistent with a scale-free network. **(e, f) **The distribution of the average clustering coefficient (C) for degree k for the entire two-hybrid dataset (e) and the high confidence set (f). C is equal to the number of interactions among a protein's interactors as a fraction of all possible interactions. **(g) **Frequency of pathlength (the shortest distance in interactions between two nodes) for the entire dataset. **(h) **Frequency of pathlength for the high confidence data.

The entire dataset of *C. jejuni *interactions and the subset of higher confidence interactions each assemble primarily into single large network components containing 99% and 95% of their interactions, respectively (Figure 1). Both networks have characteristics similar to those observed for other large-scale protein interaction datasets (Additional data file 5). Global analysis of the connectivity (k) of each protein, also known as a protein's degree, revealed a network in which most proteins have few connections, some (hubs) have many connections, and the distribution of interactions per protein is nonrandom (Figure 4a,b). A rank-degree plot of the CampyYTH v3.1 data is best modeled by an exponential curve rather than the power law expected for a scale-free network [29] (Figure 4c). In many studies, the process of selecting the higher confidence interactions has involved removal of the most highly connected proteins, or in our case, trimming interactions preferentially from those proteins. While this enriches for biologically relevant true positives, it may also change the topology of the network. Consistent with this, the higher confidence *C. jejuni *network appears to be scale-free (Figure 4d).

Several studies have shown that highly interconnected regions of experimentally derived protein interaction maps correspond to biologically relevant protein modules, such as complexes or pathways. Proteins with related functions, for example, tend to be clustered into highly interconnected subnetworks [25,30,31]. Moreover, interactions within more highly interconnected regions of protein networks tend to be enriched for true positives [32,33]. This suggests that clustering is a biological feature of a protein interaction map. The *C. jejuni *protein network has many groups of highly interconnected proteins, as indicated by its average clustering coefficient (0.10), which is high compared to other large-scale interaction maps (Additional data file 5). The *C. jejuni *higher confidence set, for example, is more highly clustered than the *Drosophila *interaction map (average clustering coefficient of 0.05 versus 0.02, respectively) even though the average number of interactions per protein in the two maps is similar. This could be explained by the fact that the *C. jejuni *map covers much more of the proteome than the *Drosophila *map. Indeed, among all the maps there is a general trend of increased clustering as the coverage increases (Additional data file 5).

### Cross-species protein interaction network conservation

**Figure 5:**
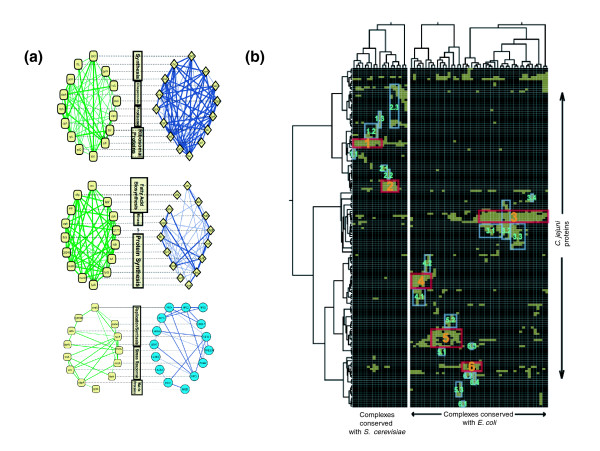
Identification of conserved core subnetworks. **(a) **Representative examples of subnetworks conserved between two organisms. *C. jejuni *subnetworks are on the left. The top and middle subnetworks (#142 and #307 in Additional data file 6) are conserved with *E. coli*. The bottom subnetwork (#56) is conserved with yeast *S. cerevisiae*. Bold lines represent direct interactions, whereas thin lines represent indirect interactions that are direct in the comparison organism (that is, these are predicted interactions). Gene names can be read by zooming in. A complete list of conserved subnetworks between *E. coli *and *S. cerevisiae *is available for download at [73]. **(b) **Hierarchical clustering of the conserved subnetworks. In the clustergram, rows represent proteins and columns represent *C. jejuni *subnetworks that are conserved with either yeast (left) or *E. coli *(right). Cores (boxed in red) and modules (boxed in blue) are defined as groups of proteins with similar profiles of subnetwork membership. The cores and modules are enriched for specific functions, for example: Core 1, serine family amino acid metabolism; Module 1-1, serine family amino acid biosynthesis; Module 1-2, generation of precursor metabolites and energy; Module 1-3, oxygen and reactive oxygen species metabolism. Larger versions of this figure are available in the Additional data files, including complex and protein names (Additional data file 8) and a list of function enrichments (Additional data file 9).

We compared the *C. jejuni *protein interaction network to protein networks from *E. coli*, *H. pylori*, and *S. cerevisiae *using the NetworkBlast algorithm, which can identify subnetworks that are conserved among species (Materials and methods) [34]. The algorithm identified 48 conserved subnetworks between *C. jejuni *and *E. coli*, and 19 between *C. jejuni *and *S. cerevisiae*. Representative conserved subnetworks are shown in Figure 5a. The subnetworks were found to be statistically significant compared to a random distribution generated by the NetworkBlast algorithm (Additional data file 6). Most of the conserved subnetworks were enriched for proteins with specific GO functions (Additional data file 6), suggesting that they represent important functional pathways or protein complexes. Surprisingly, comparison of *C. jejuni *and *H. pylori*, two organisms from the same order, resulted in no significant conserved subnetworks. This is possibly a result of low interactome coverage in the *H. pylori *protein-protein interaction network relative to the others (0.93 interactions per protein in *H. pylori *versus 1.47, 2.44, or 9.52 interactions per protein in the entire proteome of *E. coli*, *S. cerevisiae*, or *C. jejuni*, respectively). Furthermore, the fraction of the genome covered by the interaction networks differs markedly between species. Because the NetworkBlast algorithm identifies densely conserved regions of protein networks, sparse regions conserved between *H. pylori *and *C. jejuni *would not have been detected. Further analysis of the conserved subnetworks in this study allowed the prediction of a total of 379 new *C. jejuni *protein interactions (Additional data file 7). These interactions were not present in the experimental yeast two-hybrid analysis, but were derived from the significant conserved subnetworks based on the presence of the orthologous interactions in *E. coli *or *S. cerevisiae *(Materials and methods). Such predictions have become a powerful way to construct more complete interaction maps using incomplete experimental data [34,35].

To explore the potential relationships among conserved subnetworks, we used hierarchical clustering to group proteins by their subnetwork memberships (Figure 5b). These clusters support the idea, previously argued by Gavin *et al*. [3], that the network is composed of a set of functional 'cores' that interact with interchangeable 'modules' to constitute distinct cellular functions. Both cores and modules appear as groups of proteins with similar profiles of subnetwork membership; however, while core proteins appear in many subnetworks, modules appear in relatively few. Moreover, cores may appear in the presence or absence of multiple modules, whereas modules are generally found only in the presence of a particular core. These data suggest a higher level of organization amongst protein interactions within organism-wide interaction networks. Additionally, hierarchical clustering also reveals that the conserved portion of the *C. jejuni *protein-protein interaction network generated from the comparison of *C. jejuni *and *E. coli *is distinct from that generated by the comparison of *C. jejuni *and *S. cerevisiae*. This may reflect key differences in divergence between the prokaryotes *C. jejuni *and *E. coli *versus the eukaryote *S. cerevisiae*.

### A framework for protein function predictions and pathway mapping

Examination of proteins in the *C. jejuni *map that have been assigned a function (for example, based on sequence similarity to characterized proteins) reveals that proteins involved in the same process tend to interact with each other more frequently than expected by chance (Additional data file 10). This is consistent with the idea that interacting proteins in the map often function in the same pathway or protein complex. The *C. jejuni *interaction map, therefore, can be used to predict the biological role of uncharacterized proteins based on the functions of interacting proteins, as demonstrated for eukaryotic protein networks [30]. An analysis of proteins involved in flagellum biosynthesis provides a useful example. The *C. jejuni *interaction map includes an interaction between FliS, a putative flagellum assembly export chaperone, and FlaA and FlaB, the flagellin subunits comprising the flagellum. This is consistent with orthologous protein interactions detected in *Salmonella typhimurium *[36], and in the solved *Aquifex aeolicus *co-crystal structure of FliS in complex with a FliC (flagellin) fragment [37]. Unique to our *C. jejuni *dataset, however, is the additional interaction detected between FliS and the secreted protein FlaC. Despite homology to FlaA and FlaB at the amino and carboxyl termini, FlaC is not a component of the flagellum, but rather may have a role in cell invasion [38]. Experimental data indicate that the flagellar apparatus is required for secretion of FlaC [38]. Our interaction data suggest that FliS may help mediate FlaC export. The map likewise connects 663 other poorly characterized proteins into networks that provide initial clues about their functions (Figure 1).

**Figure 6:**
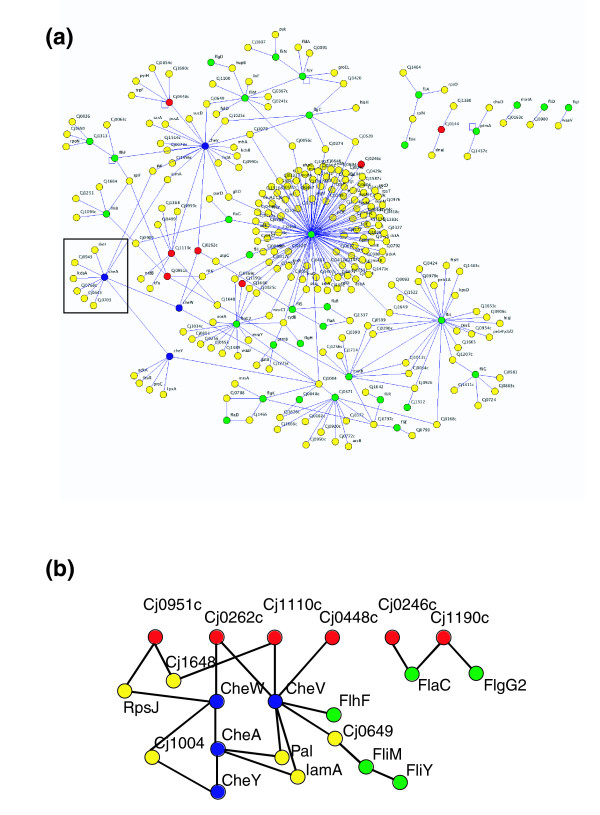
Identification of the *C. jejuni *motility protein network. **(a) **The subset of high confidence interactions involving all proteins annotated [26] as having roles in motility. Only six small networks fall outside of the single large network. Protein colors are as follows: blue, putative chemotaxis proteins; red, putative methyl-accepting chemotaxis proteins (MCPs); green, putative flagellar/motility proteins; and yellow, proteins not annotated as motility-related. The box highlights CheA and its interactors, including Cj0643 (see text). Gene names can be read by zooming in. **(b) **A subsection of the motility network highlighting the proteins in the canonical chemotaxis signal transduction pathway (MCP, CheW, CheA, CheY, and FliM) and their interactors. Proteins are colored as in (a), above. To improve visibility of interactions comprising the chemotaxis backbone, nodes not previously identified as related to chemotaxis or motility (yellow) were removed if they connected to only one red, blue, or green node.

The *C. jejuni *protein interaction dataset can also serve as a framework for mapping functional pathways, such as the chemotaxis signaling pathway (Figure 6a,b). Although not well characterized in *C. jejuni*, orthologs have been identified for the prototypical chemotaxis proteins CheW, CheA, CheY, and FliM [26,39]. In the canonical pathway, chemoattractants bind chemoreceptors known as methyl-accepting chemotaxis proteins (MCPs), which then activate the histidine kinase CheA in a complex stabilized by CheW. CheA phosphorylates CheY, which then interacts with the FliM protein at the base of the flagellar motor, resulting in changes in the direction of flagellar rotation. A search of the *C. jejuni *map for interactions involving motility and chemotaxis-related proteins reveals a large connected subnetwork of proteins (Figure 6a). The subnetwork includes the expected interactions between a putative MCP (Cj0262c) and CheW, CheW and CheA, and CheA and CheY (Figure 6b). The interaction between CheY and FliM, however, was missed, most likely because it depends upon CheY phosphorylation on a specific aspartate residue [40,41], a modification unlikely to be provided by yeast. We also identified interactions between the poorly characterized CheV protein, and three putative MCP proteins, Cj0262c, Cj0448c, and Cj1110c, supporting previous suggestions that CheV may function early in the signal transduction pathway, similar to CheW [39,42]. Lastly, we detected an interaction between CheA and Cj0643 (Figure 6a). This interaction was predicted previously [43] because Cj0643 contains the conserved CheY-like receiver domain. Cj0643 also contains a diguanylate-cyclase domain, indicating the potential for 3',5'-cyclic diguanylic acid (cdiGMP) biosynthetic activity [44]. CdiGMP is a signaling molecule in some bacteria [44]. Perhaps in *C. jejuni *the interaction between CheA and Cj0643 links cdi-GMP generation to conditions outside of the cell.

### A network of putative essential genes

**Figure 7:**
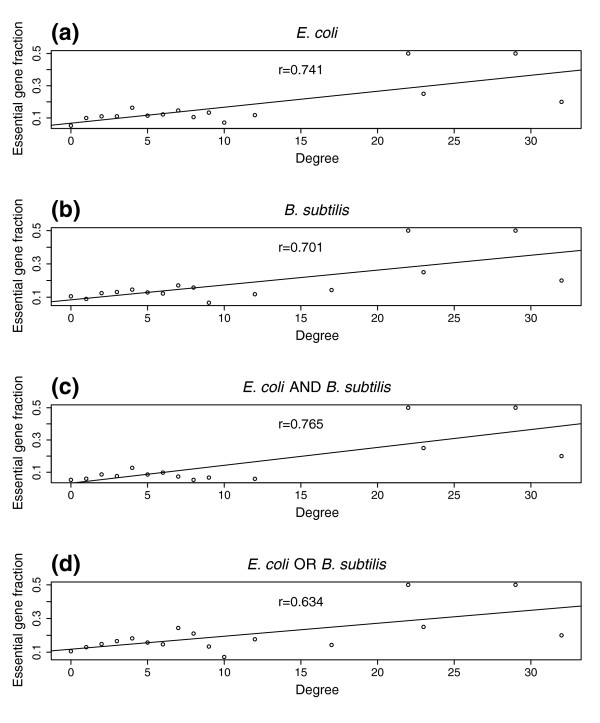
Fraction of putative *C. jejuni *essential genes among genes of the same degree. *C. jejuni *genes in the higher confidence interaction map (confidence score > 0.5) were collected into different groups according to their degrees (number of interacting partners). For each degree group the fraction of putative essential genes was computed and plotted as shown. Solid lines in the graphs were fitted using the available data points. The r-values represent Pearson correlation coefficients between fractions of putative essential genes and their degrees. **(a) **Putative *C. jejuni *essential genes are orthologs of *E. coli *genes identified as essential by Baba *et al*., [76]. **(b) **Putative *C. jejuni *essential genes are orthologs of *B. subtilits *genes identified as essential by Kobayashi *et al*., [75]. **(c) **Putative *C. jejuni *essential genes are the intersection of genes predicted to be essential from the *E. coli *and *B. subtilis *sets. **(d) **Putative *C. jejuni *essential genes are the union of the *E. coli *and *B. subtilis *sets.

Several groups have shown that in yeast, essential genes, which are genes required for growth or viability, are more likely to encode hubs in the protein network than nonessential genes [45-47]. To explore the relationship between essential genes and protein interactions in the *C. jejuni *network we generated a list of putative essential *C. jejuni *genes based on orthology to genes proposed to be essential in *E. coli *and *Bacillus subtilis *based on experimental evidence in those organisms (Materials and methods). We found higher percentages of putative essential genes amongst proteins with larger numbers of interactions (Figure 7; see also Materials and methods). It follows from this finding that, like in yeast, hub proteins are more likely to be essential than non-hub proteins. Thus, network topology may provide one way to estimate the potential importance of particular genes and may be useful in searches for new candidate drug targets.

**Figure 8:**
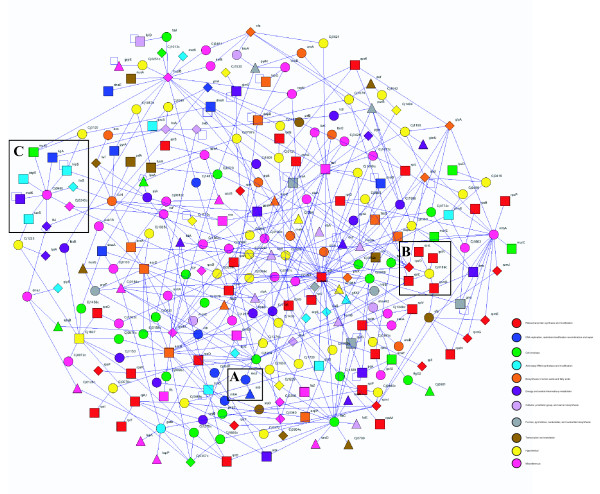
A *C. jejuni *network enriched for putative essential proteins. The network contains *C. jejuni *orthologs of genes proposed to be essential in *E. coli *(triangles), *B. subtilis *(diamonds), or in both organisms (rectangles). Additional proteins (circles) were included only if they interacted with more than one of the putative essential proteins. All of the protein interactions shown have confidence scores > 0.5. The map contains 264 proteins and 480 interactions. Proteins are colored based on their functional classification [26]; red, ribosomal protein synthesis and modification; blue, DNA replication, restriction/modification, recombination and repair; green, cell envelope; turquoise, aminoacyl tRNA synthetase and modification; orange, biosynthesis of amino acids and fatty acids; purple, energy and central intermediary metabolism; lavender, cofactor, prosthetic group and carrier biosynthesis; gray, purines, pyrimidines, nucleosides and nucleotide biosynthesis; brown, transcription and translation; yellow, hypothetical; pink, miscellaneous. Gene names can be read by zooming in.

Essential proteins often function together in pathways or processes that are important for cell growth or viability. Consistent with this, we found that the *C. jejuni *map contains interactions between putative essential proteins significantly more frequently than expected by chance (Additional data file 11). Similar results have been described for yeast protein interaction maps [46]. One consequence of this enrichment for essential-essential interactions is that groups of essential proteins can form interconnected subnetworks within the interaction map. Additionally, the *C. jejuni *map may be used to predict that some of the previously uncharacterized proteins may be important for growth or viability based on their interactions with known essential proteins. To create a network enriched for important proteins we identified a subnetwork of interconnected proteins predicted to be essential in *C. jejuni *based on orthology to essential proteins in *E. coli *and *B. subtilis *(Figure 8, triangular, diamond, and rectangular nodes). To identify additional putative essential or important proteins, we added proteins that connect to two or more of the essential nodes through high confidence interactions (circular nodes). The resulting map (Figure 8) contains 264 proteins, many of which are of unknown function (yellow), and identifies potential connections amongst many proteins involved in processes known to be essential for viability, including ribosome function and DNA synthesis and repair. For example, Box A in Figure 8 highlights the interaction between RecJ and SSB. SSB is a single-stranded DNA (ssDNA) binding protein that resolves secondary structure in ssDNA (reviewed in [48]), while RecJ is a conserved exonuclease that degrades ssDNA [49]. Both proteins have roles in homologous recombination and mismatch repair [48,50,51]. A recent report has demonstrated that binding of ssDNA by SSB enhances RecJ binding and exonuclease activity [52], suggesting a functional relationship between the two proteins. This is further supported by the binary protein-protein interaction that we have detected in *C. jejuni *(this study) and the purification of an *E. coli *protein complex containing RecJ using affinity-tagged SSB [1].

The many uncharacterized proteins in the essential protein network are potentially biologically important and may include potential novel drug targets. For example, Box B in Figure 8 highlights a protein of unknown function, Cj0189c, which has interaction partners with five ribosomal proteins. Based on this and the fact that proteins with related functions tend to interact, it is reasonable to hypothesize that Cj0189c may also be involved in ribosome assembly or function. This is potentially significant given that the ribosome and protein synthesis are frequent targets of antibiotics [53]. Box C in Figure 8 highlights the uncharacterized protein Cj0980, which is homologous to the dipeptidase, peptidase D. In *E. coli*, peptidase D is one of the enzymes that generates cysteine by cleaving cysteinylglycine [54]. In our map, Cj0980 interacts with nine proteins predicted to be essential. One of these proteins, Cj0240c, is a homolog of IscS, a cysteine desulfurase required for the synthesis of all tRNA thiolated nucleosides in *E. coli *[55]. Interestingly, four additional interactors of Cj0980 are tRNA synthetases. Whether or not their product tRNAs are modified in *C. jejuni *has not been determined, but this series of interactions suggests a possible pathway or protein complex that mediates the transfer of a thiol group originating from cysteinylglycine to specific tRNAs.

## Discussion

The large-scale interaction studies performed to date have fallen short of complete interactome coverage. The most complete large-scale yeast two-hybrid screens have covered only around 54% of the proteome in *Drosophila *[22,25,56], 46% in *H. pylori *[11] and 55% in yeast [57-59], while co-AP/MS studies have reached 80% and 67% of the *E. coli *and yeast proteomes, respectively [1-6] (Additional data file 1). Complete interactome coverage should include most of the proteome, since most proteins are believed to function at least in part through interactions with other proteins. A major factor contributing to incomplete coverage is the incomplete nature of the high-throughput screens, as indicated by the minimal rate of overlap observed between independent large-scale screens (Additional data file 1) [22,59]. Thus, despite the usefulness of the data from various interaction mapping efforts, the low interactome coverage is likely to limit efforts to predict protein functions, map pathways, and characterize protein networks. Low coverage also limits the opportunity for cross-validation, which is particularly important for high-throughput datasets because they tend to have high rates of false positives [24,60].

We have made substantial progress towards defining the *C. jejuni *interactome. Based on the number of ORFs included in the interaction dataset, we have covered 80% of the proteome, and our higher confidence dataset covers 67%. An expected consequence of performing high-throughput screens, which tend to be subsaturating, is that some interactions that are detectable by two-hybrid assays are missed [10]. We set out to minimize these false negatives by using a highly sensitive two-hybrid system, inducible promoters to detect interactions with toxic proteins and transcriptional activators, and a pooled-matrix mating scheme to maximize the number of interactions sampled. Despite these efforts, some interactions will be missed, especially those that are refractory to standard two-hybrid assays. Detection of these will require other technologies, such as isolation and identification of protein complexes, and assays that target specific classes of proteins, such as membrane proteins [61,62]. Interaction networks may also be made more complete by using computational approaches to predict missed interactions [34,35]. In this study we applied a comparative algorithm to align protein networks from *C. jejuni *to the interactomes of other species to generate further predictions of protein interactions. Like the high throughput experimental data, these predictions provide a guide for directed validation studies.

An unfortunate side effect of large-scale protein interaction datasets is the presence of significant numbers of false positive interactions. We addressed this problem in two ways. First, we retested every interaction in a second independent two-hybrid assay. Second, we calculated probability scores that correlate with the likelihood that an interaction is biologically relevant. One advantage to this confidence scoring system is that it scores interactions rather than proteins and, therefore, does not specifically delete any proteins. Several studies, including ours, have found an inverse correlation between the biological significance of an interaction and the total number of interactions for the two proteins involved; the more interactions that a protein has, the less likely they are to be biological true positives. One approach to increasing the overall confidence of a dataset, therefore, is to delete these 'sticky' proteins. In contrast, it is possible to identify biologically relevant interactions involving these proteins by using a statistical scoring system that weighs multiple attributes according to their correlation with biological significance. With such a scoring system an interaction may be penalized because it involves a sticky protein, but redeemed due to some other attribute. This is the case, for example, in our data with the interactions FliS-FlaC, GroEL-GroES, Ilvl-IlvH, PyrB-PyrC2, and TrxA-TrxB, all of which involve proteins with more than 60 interactions, yet have confidence scores above 0.8, and are likely to be biologically significant.

Another advantage to this scoring system is that it allows user-defined confidence intervals to be chosen based on particular analysis needs. Global analyses, for example, may benefit from using the highest confidence dataset. More focused analyses involving one or few proteins, on the other hand, may tolerate lower confidence interactions because validation experiments can be performed. This reduces the chances of missed interactions. Importantly, some low confidence interactions may be found to be biologically significant by experimental validation or by considering additional information not used in the scoring system. For example, by considering pairs of proteins with known functions, one can find a number of likely true positives with confidence scores below 0.2, including DnaX-DnaN, ExbD1-ExbD3, and FabF-FabG.

Finally, the confidence that we have in any particular interaction can change as new data become available about the two proteins or about the interaction itself. We have shown that the scores we assigned to the *C. jejuni *two-hybrid data correlate with biological significance such that more of the interactions with higher scores will be biologically significant than those with lower scores, and vice versa. Nevertheless, a fraction of the low confidence interactions are true positives and some of the high confidence interactions are false positives. It is expected that these will be sorted out using new, increasingly accurate confidence scoring systems that are based, for example, on new information as it becomes available. Thus, we have defined the scoring of the *C. jejuni *two-hybrid data presented here as version 1.0.

## Conclusion

Interactome maps such as the one generated in our study begin to provide a tally of the binary protein interactions that can occur within an organism. Although incomplete, the data can provide a framework for understanding dynamic biological processes, such as the *C. jejuni *chemotaxis response. The map also can be mined for subnetworks of biological interest, such as essential gene networks that suggest candidate drug targets. Comparative analyses of protein interaction maps generated for humans and model eukaryotes have provided insights into the function and evolution of proteins and their regulatory networks. The protein interactions detected for each species also have enabled the prediction of interactions in other species, which is particularly important given the difficulty of obtaining complete coverage in high throughput screens, and the lack of suitable screening systems for many species. The *C*. *jejuni *interaction map generated here substantially increases the protein interactions detected thus far for the prokaryotic domain of life. The map should provide a useful starting point for predicting the functions of uncharacterized proteins and for mapping functional pathways in *C. jejuni *and other prokaryotes.

## Materials and methods

### Strains and plasmids

The two-hybrid system used here is based on the version originally described by Brent and colleagues [63]. *C. jejuni *ORFs were cloned into the yeast two-hybrid vector pJZ4-NRT for expression of AD fusions driven by the yeast *GAL*1 promoter [22], and pHZ5-NRT for expression of LexA DNA BD fusions driven by the yeast *MAL*62 promoter [23]. Both vectors contain recombination tags for direct cloning of tagged inserts (see below). Yeast strain RFY231 (MATα *trp1*Δ::*hisG his3 ura3-1 leu2*::3LexAop-*LEU2*) contained the AD plasmids, while Y309 (MATa *trp1*Δ::*hisG his3*Δ200 *leu2-3 lys2*Δ201 *ura3-52 *mal- pSH18-34(*URA3*, *lacZ*)) contained the BD plasmids. The reporter genes include *LEU2*, facilitating growth on medium lacking leucine, and *lacZ*, expression of which turns yeast colonies blue when the substrate X-Gal is present.

### Generation of yeast two-hybrid arrays for *C. jejuni*

PCR amplification of over 87% of the predicted ORFs from *C. jejuni *NCTC11168 genomic DNA was previously described [64]. The amplification products included the 21 bp recombination tags 5RT1 and 3RT1 at their 5' and 3' ends, respectively, which match identical sites flanking the insertion site in the yeast two-hybrid vectors. PCR products were cloned into the vectors via homologous recombination in yeast as described previously [22]. To validate the identity of the insert in each vector, the 5' ends of the inserted PCR products were sequenced. We generated 1,398 BD strains and 1,442 AD strains containing the two-hybrid vectors with inserts, of which 90% have been sequence verified. Most of the ORFs missing from the arrays failed PCR amplification prior to cloning.

### High-throughput yeast two-hybrid analysis

We mated BD and AD strains using a two-phase pooling (pooled matrix) strategy as described previously [21,22]. Briefly, 15 pools of approximately 96 AD strains each were generated, along with one additional pool of 32 strains. Each pool was mated with individual BD strains arrayed on 96-well plates, and the resulting diploids were assayed for reporter activities. Positive BD strains were then mated with each member of the positive AD pool arrayed on 96-well plates to identify the interacting pairs. Reporter activities were scored using a custom program for image analysis [65] and at least one manual scoring. LacZ scores ranged from 0 (white) to 5 (dark blue) and Leu scores ranged from 0 (no growth) to 3 (heavy growth); combined scores ranged from 0 to 8. Many BDs have some level of background activity due to activation independent of the AD fusion or non-specific interactions. To correct for these we calculated the average interaction score for each BD based on at least 96 interaction assays and subtracted this background from the reporter scores for each of its interactions. Of these corrected scores, only those ≥ 1 were considered initial positives and were retested (see below). A small subset of BD strains (94 total) was also assayed using a library approach as described [21,22]. Briefly, BD strains were individually mated with a single pool containing almost all of the AD strains (except Cj1718c (*leuB*) and Cj1546, which activate reporters without a BD). Up to 30 diploids with reporter activity were picked for each BD. Their AD inserts were PCR amplified and restriction digested to identify strains carrying the same clones. Single representatives from each restriction fragment class (RFC) were then sequenced to identify the inserts. Of the 134 interactions detected, 52 (39%) were also identified in the two-phase matrix screen. Combined, 16,104 unique interactions were retested in one-on-one binary mating assays between individual AD and BD strains on 96-well plates. A total of 11,687 interactions proved repeatable (background-corrected combined activity score ≥ 1), including 73% of those from the two-phase matrix screen, 75% of those from the library screen, and 100% of those detected in both screens. The majority of interactions that failed to repeat had been low-scoring (less than 2) in the initial screen. The 11,687 interactions that repeated were combined with 325 non-repeated interactions that had high confidence scores (see below) to create a dataset containing 12,012 interactions, which we named CampyYTH v3.1. This version of the dataset was subsequently used for bioinformatics analysis as indicated. The interaction data can be visualized and downloaded at [17]. The CampyYTH v3.1 data are also listed in Additional data file 13.

### Assignment of confidence scores

Confidence scores were determined for each interaction based on methods described by Bader and colleagues [24,25]. We fit a generalized linear model [66] using experimental and topological attributes of yeast two-hybrid interactions, including the number of interactions for each protein in a pair and the Leu and *lacZ *reporter activities Fitting the model required both positive and negative training sets. Because a reference set of known interactions is not available for *C. jejuni*, we derived a set of positive training data (85 interactions total) by assuming that the conserved interactions (reciprocal best match interologs) in common with either the *E. coli *low-throughput interaction set [28], the *H. pylori *yeast two-hybrid set [11], or the *E. coli *protein complex set [1] are likely to be true positives. We derived a set of likely true negatives (111 total) for the negative training data by considering interactions between proteins whose orthologs in *E. coli *or *H. pylori *were separated in the respective interaction maps by greater than the average distance of all pairs (≥ 4). Positive and negative training cases were weighted inversely to the number of interactions in each set. When training sets are weighted this way, a confidence score greater than 0.5 means that available data and features support that a specific interaction has a better than random chance to be a true interaction; this allows 0.5 to be used as the threshold between high and low confidence interactions. Validation using protein features not used in the scoring system support the choice of 0.5 as a threshold for higher confidence interactions (discussed further in Additional data file 14; see also Figure 2c). Of the attributes tested, the numbers of interactions per protein were found to be negative predictors of biologically relevant interactions, while reporter activities were positive predictors. To evaluate the scoring model, we performed a stratified five-fold cross-validation. Cross-validation reported a precision of 91.4% and a recall of 78.9%, which gave us confidence that it is a reasonably well-fitted model. We then used the full sets of positives and negatives in training and obtained our final logistic model. The final model was used to compute confidence scores for 16,104 initial positive interactions prior to retesting. Of these, 3,209 scored higher than 0.5, which we define as the high confidence set. Of the interactions with high confidence scores (> 0.5), 90% corresponded to interactions that repeated when retested, while only 68% of the low confidence interactions repeated. Further discussion and details of the confidence scoring system are available in Additional data file 14.

### Evaluating the confidence score model

Main role annotations 'mainrole' were downloaded from [67]. Excluding self-interactions, out of the 3,209 high confidence interactions, 2,599 have 'mainrole' annotations, and 454 share at least one 'mainrole' annotation. We generated 5,000 groups of 2,599 randomly selected interactions that have 'mainrole' annotations and have a confidence score lower than 0.5. The number of pairs in each set that share 'mainrole' annotations was counted. The distribution was plotted in a histogram and compared with the high confidence set (Figure 2b). To examine whether high confidence interactions tend to share more detailed GO [27] annotations, we grouped interactions into confidence bins so that each bin contains only interactions with scores falling into a specific range. For each interaction, we determined the deepest level of GO biological process annotations shared by the pair of genes, and calculated the average depth of shared biological process for each group. Since GO for *C. jejuni *NCTC11168 was not available, we used annotations for best match orthologs of *C. jejuni *RM1221 genes [68]. Figure 2c shows that there is a general pattern of increased depth of shared GO terms for interactions with confidence score higher than 0.5. This fact also suggests that our choice of 0.5 as a high confidence threshold is meaningful.

### Assessment of functional enrichments

The frequency of each GO description from the iProClass database [69], amongst all of the proteins comprising the proteome was determined and compared to their frequency within the CampyYTH v3.1 dataset or the high confidence subset (Additional data file 3). A similar analysis was performed using the functional classifications assigned by the Sanger Institute [26] (Additional data file 2). We also looked for pairs of GO annotations that were enriched in the interaction data (Additional data file 10). To do this we counted the number of interactions having a specific pair of GO terms. We mapped the annotations to level 5; that is, for a protein with GO annotation A that is at a deeper level than 5, we mapped A to level 5 using 'parent' and 'part of' relationships in the ontologies, and we discarded A if it was above level 5. Self-interactions were excluded from the analysis. We did the same for all GO terms annotated to a protein. To compute the significance of finding specific GO pairs, we generated 2,000 random networks by randomly switching pairs of links while maintaining the degree distribution of the original map, and counted the number of times we found each GO pair in each randomized network. For each GO pair, a *p *value was computed based on the distribution of the 2,000 counts (assuming normal distribution) and the count in the original yeast two-hybrid map. The *p *value represents the probability of seeing such a pair in a random network. We listed only pairs with a *p *value less than 5%.

### Comparative network analysis

Additional details are in Additional file 14. Protein-protein interactions from *C. jejuni *were compared with those from *E. coli *[1], *H. pylori *[11] and *S. cerevisiae *from DIP [28]. Corresponding protein sequences were obtained from the following sources: *C. jejuni *NCTC11168 [26]; *E. coli *[70]; *H. pylori *[71]; and *S. cerevisiae *[72]. We used NetworkBlast to identify significant conserved protein-protein interaction subnetworks [34]. A stand-alone Java version of the program is available at [73]. Briefly, the algorithm takes as input a pair of protein-protein interaction networks, one for each of two species, along with a set of homology relationships between the proteins of the two networks. We constructed the homology relationships from an all-versus-all BLAST of the complete set of protein sequences for each of the two species, taking the top 10 hits with E-value = 10^-10^. Next, a network alignment graph was created where each node represents a homologous pair of proteins from species 1 and 2 (for example, a1 and a2) and each edge represents a conserved interaction (a1/a2 connects to b1/b2 if the a-b interaction is found in both species; interactions may be either direct (distance 1) or indirect (distance 2), in which a-b is connected through a common neighbor, that is, a-c-b). A greedy search is initiated from each node to identify conserved protein subnetworks, defined as dense subgraphs within the network alignment graph (of maximum size 15 proteins per species). When multiple subnetworks contain protein homologs that overlap by ≥ 50%, only the complex with the highest density was included in the final result. GO annotations [27] of proteins in each conserved complex were analyzed to identify significant functional enrichments (Additional data file 6). We calculated a hypergeometric *p *value of enrichment for each GO annotation in the three divisions of the GO hierarchy and constrained the annotations by requiring that at least half of the proteins in a complex ascribe to the enrichment. The most specific annotations with hypergeometric *p *value < 0.05 in each of the three divisions were then assigned to each complex. A complete list of conserved complexes between *C. jejuni *and *E. coli *or *S. cerevisiae *is available for download at [73]. The significant conserved subnetworks provided predictions of 379 new *C. jejuni *protein-protein interactions not found in the two-hybrid screens (Additional data file 7). A protein pair (a, b) was predicted to interact directly if: first, both a and b were present in the same significant conserved complex; second, this pair was observed to interact indirectly in *C. jejuni*; and third, this pair corresponded to a direct interaction in the comparison species' network.

### Clustering of conserved subnetworks

Since proteins can belong to more than one complex, we clustered the significant conserved subnetworks by protein membership, in effect 'superclustering' the interactions (Figure 5b). An *n *× *m *matrix was constructed, where *n *is the number of significant subnetworks and *m *is the number of unique proteins involved in any of the significant subnetworks. Using the open source tool ClustArray [74], we clustered the proteins hierarchically using the unweighted pair group method with arithmetic mean (UPGMA) and clustered the subnetworks with a combination *k*-means algorithm followed by UPGMA hierarchical clustering. The number of clusters *k *= *3 *was chosen as the parameter that approximately minimized within-cluster variability and maximized between-cluster variability (data not shown). Identities of complexes and proteins are shown in the high resolution image of the hierarchical clustering in Additional data file 8. Lists of the proteins comprising complexes are available for download at [73].

### Essential gene analysis and network assembly

We generated lists of putative *C. jejuni *NCTC11168 essential proteins by identifying reciprocal best match orthologs of likely essential proteins from *B. subtilis *[75] and *E. coli *[76]. We removed genes from our putative essential list if viable null mutants have been reported (Dr. B. Wren, personal communication). To examine the relationship between essentiality and centrality in the interaction map, we computed the numbers of essential and non-essential proteins in groups having the same number of interactions (degree) in the higher confidence dataset (interactions with confidence scores > 0.5). The result is shown in Figure 7, where r values in the graphs represent Pearson correlation coefficients between the fractions and the degrees. Figure 7 shows that there is a correlation between degree of proteins and the likelihood of being essential. A similar result was obtained with the entire dataset CampyYTH v3.1 (not shown). Lastly, we computed the fraction of essential and non-essential neighbors of each essential protein and compared this to the fraction for random groups of proteins (of the same size as the set of essential proteins). The results shown in Additional data file 11 indicate that essential genes tend to have more neighbors that are also essential; *p *values indicate the probability of seeing the real fraction (the red dot) by chance.

## Additional data files

The following additional data are available with the online version of this paper. Additional data file 1 is a table summarizing proteome coverage from large-scale interaction screens. Additional data file 2 is a table listing the representation of functional categories amongst the proteins in the CampyYTH v3.1 dataset. Additional data file 3 is a table listing the GO category representation amongst the proteins in CampyYTH v3.1. Additional data file 4 lists *C. jejuni *genes that were toxic or inhibitory to yeast growth. Additional data file 5 is a table comparing network features across organisms. Additional data file 6 lists conserved subnetworks between *C. jejuni *and *E. coli *or *C. jejuni *and yeast. Additional data file 7 lists predicted *C. jejuni *protein interactions. Additional data file 8 is a higher resolution version of Figure 5, showing hierarchical clustering of conserved subnetworks. Additional data file 9 is a table listing enriched functions within the cores and modules of Figure 5. Additional data file 10 is a table showing GO enrichment amongst the *C. jejuni *protein interactions. Additional data file 11 is a figure showing that essential proteins interact with each other more often than expected by chance. Additional data file 12 is a table of *C. jejuni *interologs predicted from large-scale protein interaction analyses performed for *E. coli *or *H. pylori*. Additional data file 13 is an annotated list of all *C. jejuni *protein interactions in the CampyYTH v3.1 dataset. Additional data file 14 includes supplementary materials and methods.

## Supplementary Material

Additional data file 1 Proteome coverage from large-scale interaction screens Click here for file

Additional data file 2 Representation of functional categories amongst the proteins in the CampyYTH v3.1 dataset Click here for file

Additional data file 3 GO category representation amongst the proteins in CampyYTH v3.1 Click here for file

Additional data file 4 *C. jejuni *genes that were toxic or inhibitory to yeast growth Click here for file

Additional data file 5 Comparison of network features across organisms Click here for file

Additional data file 6 Conserved subnetworks between *C. jejuni *and *E. coli *or *C. jejuni *and yeast Click here for file

Additional data file 7 Predicted *C. jejuni *protein interactions Click here for file

Additional data file 8 Higher resolution version of Figure 5, showing hierarchical clustering of conserved subnetworks Click here for file

Additional data file 9 Enriched functions within the cores and modules of Figure 5 Click here for file

Additional data file 10 GO enrichment amongst the *C. jejuni *protein interactions Click here for file

Additional data file 11 Essential proteins interact with each other more often than expected by chance Click here for file

Additional data file 12 *C. jejuni *interologs predicted from large-scale protein interaction analyses performed for *E. coli *or *H. pylori* Click here for file

Additional data file 13 Annotated list of all *C. jejuni *protein interactions in the CampyYTH v3.1 dataset Click here for file

Additional data file 14 Supplementary materials and methods Click here for file
