# Supplementary material for: A proteome-wide protein interaction map for Campylobacter jejuni
Source: Genome Biol. 2007 Jul 5;8(7):R130. doi: 10.1186/gb-2007-8-7-r130 (PMC2323224; doi:10.1186/gb-2007-8-7-r130)
Supplement: Additional data file 1 — Proteome coverage from large-scale interaction screens [file gb-2007-8-7-r130-S1.doc]

**Additional Data File 1. Summary of proteome coverage from large-scale interaction screens.**

| **Organism** | **Genes** | **Studies** | **Method^1^** | **Genes repre-sented^2^** | **Coverage (%)** | **Interactions detected^3^** | **Gene intersect^4^** | **Interaction intersect^4^** | **References** |
| --- | --- | --- | --- | --- | --- | --- | --- | --- | --- |
| *Campylobacter jejuni* NCTC11168^5^ | 1,654 | 1 | YTH | 1321 | 79.9 | 11,687 | NA | NA | This study |
| *Helicobacter pylori* 26695 | 1,587 | 1 | YTH | 732 | 46.1 | 1,465 | NA | NA | [1] |
| *Pyrococcus horikoshii shinkaj* OT3 | 2,064 | 1 | MTH | 172 | 8.3 | 170 | NA | NA | [2] |
| *Caenorhabditis elegans* | 22,841 | 1 | YTH | 2847 | 12.5 | 4,584 | NA | NA | [3] |
| *Plasmodium falciparum* | 5,300 | 1 | YTH | 1308 | 24.7 | 2,811 | NA | NA | [4] |
| *Drosophila***^6^** | 13,987 | 3 | YTH | 7494 | 53.6 | 24,121 | 94 | 1 | [5-7] |
| Human | 24,000 | 2 | YTH | 2998 | 12.5 | 5,922 | 256 | 10 | [8, 9] |
| *Escherichia coli* K12 | 4,289 | 2 | CoAP/MS | 3440 | 80.2 | 16,559 | 980 | 174 | [10, 11] |
| Yeast (CoAP/MS)**^6^** | 6,593 | 3 | CoAP/MS | 4441 | 67.4 | 35,223 | 611 | 60 | [12-15] |
| Yeast (YTH)**^6^** | 6,593 | 3 | YTH | 3645 | 55.3 | 5,639 | 887 | 202 | [16-18] |
| Yeast (combined) | 6,593 | 6 | CoAP/MS & YTH | 5418 | 82.2 | 40,531 | 142 | 2 | See above |

^1^Abbreviations are as follows: YTH, yeast two-hybrid; MTH, mammalian two-hybrid; CoAP/MS, protein complex purification/mass spectrometric identification.

**^2^**The number of genes represented in the interaction datasets. For organisms with more than one study, the protein union is reported (i.e., proteins in common amongst the studies

are counted only once).

**^3^**For organisms with more than one study, the interaction union is reported (i.e., interactions detected in more than one study are counted only once.)

**^4^**Gene intersect refers to the number of genes in common between multiple studies for the same organism. Likewise, interaction intersect refers to the number of protein interactions

in common.

**^5^**The CampyYTH v3.1 dataset repeatable interactions were used.

^6^Yeast interactions were obtained from DIP [19], [14], and [15]. *Drosophila* interactions were obtained from the *Drosophila* Interactions Database [20].

**References:**

1. Rain JC, Selig L, De Reuse H, Battaglia V, Reverdy C, Simon S, Lenzen G, Petel F, Wojcik J, Schachter V *et al*: **The protein-protein interaction map of *Helicobacter pylori****.* *Nature* 2001, **409**(6817):211-215.

2. Usui K, Katayama S, Kanamori-Katayama M, Ogawa C, Kai C, Okada M, Kawai J, Arakawa T, Carninci P, Itoh M *et al*: **Protein-protein interactions of the hyperthermophilic archaeon *Pyrococcus horikoshii* OT3**. *Genome Biol* 2005, **6**(12):R98.

3. Li S, Armstrong CM, Bertin N, Ge H, Milstein S, Boxem M, Vidalain PO, Han JD, Chesneau A, Hao T *et al*: **A map of the interactome network of the metazoan *C. elegans***. *Science* 2004, **303**(5657):540-543.

4. LaCount DJ, Vignali M, Chettier R, Phansalkar A, Bell R, Hesselberth JR, Schoenfeld LW, Ota I, Sahasrabudhe S, Kurschner C *et al*: **A protein interaction network of the malaria parasite *Plasmodium falciparum***. *Nature* 2005, **438**(7064):103-107.

5. Giot L, Bader JS, Brouwer C, Chaudhuri A, Kuang B, Li Y, Hao YL, Ooi CE, Godwin B, Vitols E *et al*: **A protein interaction map of *Drosophila melanogaster***. *Science* 2003, **302**(5651):1727-1736.

6. Stanyon CA, Liu G, Mangiola BA, Patel N, Giot L, Kuang B, Zhang H, Zhong J, Finley RL, Jr.: **A *Drosophila* protein-interaction map centered on cell-cycle regulators**. *Genome Biol* 2004, **5**(12):R96.

7. Formstecher E, Aresta S, Collura V, Hamburger A, Meil A, Trehin A, Reverdy C, Betin V, Maire S, Brun C *et al*: **Protein interaction mapping: a *Drosophila* case study**. *Genome Res* 2005, **15**(3):376-384.

8. Stelzl U, Worm U, Lalowski M, Haenig C, Brembeck FH, Goehler H, Stroedicke M, Zenkner M, Schoenherr A, Koeppen S *et al*: **A human protein-protein interaction network: a resource for annotating the proteome**. *Cell* 2005, **122**(6):957-968.

9. Rual JF, Venkatesan K, Hao T, Hirozane-Kishikawa T, Dricot A, Li N, Berriz GF, Gibbons FD, Dreze M, Ayivi-Guedehoussou N *et al*: **Towards a proteome-scale map of the human protein-protein interaction network**. *Nature* 2005, **437**(7062):1173-1178.

10. Butland G, Peregrin-Alvarez JM, Li J, Yang W, Yang X, Canadien V, Starostine A, Richards D, Beattie B, Krogan N *et al*: **Interaction network containing conserved and essential protein complexes in *Escherichia coli***. *Nature* 2005, **433**(7025):531-537.

11. Arifuzzaman M, Maeda M, Itoh A, Nishikata K, Takita C, Saito R, Ara T, Nakahigashi K, Huang HC, Hirai A *et al*: **Large-scale identification of protein-protein interaction of *Escherichia coli* K-12**. *Genome Res* 2006, **16**(5):686-691.

12. Gavin AC, Bosche M, Krause R, Grandi P, Marzioch M, Bauer A, Schultz J, Rick JM, Michon AM, Cruciat CM *et al*: **Functional organization of the yeast proteome by systematic analysis of protein complexes**. *Nature* 2002, **415**(6868):141-147.

13. Ho Y, Gruhler A, Heilbut A, Bader GD, Moore L, Adams SL, Millar A, Taylor P, Bennett K, Boutilier K *et al*: **Systematic identification of protein complexes in *Saccharomyces cerevisiae* by mass spectrometry**. *Nature* 2002, **415**(6868):180-183.

14. Gavin AC, Aloy P, Grandi P, Krause R, Boesche M, Marzioch M, Rau C, Jensen LJ, Bastuck S, Dumpelfeld B *et al*: **Proteome survey reveals modularity of the yeast cell machinery**. *Nature* 2006, **440**(7084):631-636.

15. Krogan NJ, Cagney G, Yu H, Zhong G, Guo X, Ignatchenko A, Li J, Pu S, Datta N, Tikuisis AP *et al*: **Global landscape of protein complexes in the yeast *Saccharomyces cerevisiae***. *Nature* 2006, **440**:637-643.

16. Uetz P, Giot L, Cagney G, Mansfield TA, Judson RS, Knight JR, Lockshon D, Narayan V, Srinivasan M, Pochart P *et al*: **A comprehensive analysis of protein-protein interactions in *Saccharomyces cerevisiae***. *Nature* 2000, **403**(6770):623-627.

17. Ito T, Tashiro K, Muta S, Ozawa R, Chiba T, Nishizawa M, Yamamoto K, Kuhara S, Sakaki Y: **Toward a protein-protein interaction map of the budding yeast: A comprehensive system to examine two-hybrid interactions in all possible combinations between the yeast proteins**. *Proc Natl Acad Sci U S A* 2000, **97**(3):1143-1147.

18. Ito T, Chiba T, Ozawa R, Yoshida M, Hattori M, Sakaki Y: **A comprehensive two-hybrid analysis to explore the yeast protein interactome**. *Proc Natl Acad Sci U S A* 2001, **98**(8):4569-4574.

19. Xenarios I, Rice DW, Salwinski L, Baron MK, Marcotte EM, Eisenberg D: **DIP: the database of interacting proteins**. *Nucleic Acids Res* 2000, **28**(1):289-291.

20. Pacifico S, Liu G, Guest S, Parrish JR, Fotouhi F, Finley RL, Jr.: **A database and tool, IM Browser, for exploring and integrating emerging gene and protein interaction data for *Drosophila***. *BMC Bioinformatics* 2006, **7**(1):195.
