# Supplementary material for: A proteome-wide protein interaction map for Campylobacter jejuni
Source: Genome Biol. 2007 Jul 5;8(7):R130. doi: 10.1186/gb-2007-8-7-r130 (PMC2323224; doi:10.1186/gb-2007-8-7-r130)
Supplement: Additional data file 2 — Representation of functional categories amongst the proteins in the CampyYTH v3.1 dataset [file gb-2007-8-7-r130-S2.doc]

**Additional Data File 2.** Functional category representation amongst the proteins in the CampyYTH v3.1 dataset.

| Function Category^1^ | Function Description | Freq. in Proteome | Freq. in Proteome (%) | Freq. in v3.1 Exp | Freq. in v3.1 Exp (%) | Enriched in v3.1 (%) | Freq. in High Conf. Set | Freq. in High Conf. Set (%) | Enriched in High Conf. Set (%) |
| --- | --- | --- | --- | --- | --- | --- | --- | --- | --- |
| 1.A | Small molecule degradation | 6 | 0.36 | 5 | 0.38 | 0.01 | 5 | 0.45 | 0.09 |
| 1.B | Energy metabolism of small molecule | 103 | 6.23 | 85 | 6.38 | 0.15 | 73 | 6.59 | 0.36 |
| 1.C | Central intermediary metabolism of small molecules | 29 | 1.75 | 25 | 1.88 | 0.12 | 22 | 1.99 | 0.23 |
| 1.D | Amino acid biosynthesis | 66 | 3.99 | 55 | 4.13 | 0.14 | 52 | 4.69 | 0.70 |
| 1.E | Polyamine synthesis | 1 | 0.06 | 1 | 0.08 | 0.01 | 1 | 0.09 | 0.03 |
| 1.F | Purines, pyrimidines, nucleosides and nucleotides metabolism | 37 | 2.24 | 31 | 2.33 | 0.09 | 30 | 2.71 | 0.47 |
| 1.G | Biotin metabolism | 54 | 3.26 | 47 | 3.53 | 0.26 | 40 | 3.61 | 0.35 |
| 1.H | Fatty acid biosynthesis | 21 | 1.27 | 16 | 1.20 | -0.07 | 15 | 1.35 | 0.08 |
| 2 | Broad regulatory functions | 23 | 1.39 | 21 | 1.58 | 0.19 | 20 | 1.81 | 0.41 |
| 2.1 | Signal transduction | 25 | 1.51 | 16 | 1.20 | -0.31 | 14 | 1.26 | -0.25 |
| 3.A | Synthesis and modification of macromolecules | 202 | 12.21 | 165 | 12.39 | 0.17 | 138 | 12.45 | 0.24 |
| 3.B | Degradation of macromolecules | 22 | 1.33 | 16 | 1.20 | -0.13 | 11 | 0.99 | -0.34 |
| 3.C | Cell envelope | 396 | 23.94 | 309 | 23.20 | -0.74 | 236 | 21.30 | -2.64 |
| 4.A | Transport/binding proteins | 138 | 8.34 | 98 | 7.36 | -0.99 | 66 | 5.96 | -2.39 |
| 4.B | Chaperones, chaperonins, heat shock | 17 | 1.03 | 16 | 1.20 | 0.17 | 14 | 1.26 | 0.24 |
| 4.C | Cell division | 8 | 0.48 | 5 | 0.38 | -0.11 | 5 | 0.45 | -0.03 |
| 4.D | Chemotaxis and mobility | 9 | 0.54 | 7 | 0.53 | -0.02 | 6 | 0.54 | 0.00 |
| 4.E | Protein and peptide secretion | 15 | 0.91 | 10 | 0.75 | -0.16 | 8 | 0.72 | -0.18 |
| 4.G | Detoxification | 7 | 0.42 | 7 | 0.53 | 0.10 | 5 | 0.45 | 0.03 |
| 4.I | Pathogenicity | 13 | 0.79 | 9 | 0.68 | -0.11 | 7 | 0.63 | -0.15 |
| 5.A | IS elements | 1 | 0.06 | 0 | 0.00 | -0.06 | 0 | 0.00 | -0.06 |
| 5.C | Plasmid related functions | 1 | 0.06 | 1 | 0.08 | 0.01 | 1 | 0.09 | 0.03 |
| 5.D | Drug/analogue sensitivity | 8 | 0.48 | 3 | 0.23 | -0.26 | 3 | 0.27 | -0.21 |
| 5.F | Adaptions and atypical conditions | 2 | 0.12 | 2 | 0.15 | 0.03 | 2 | 0.18 | 0.06 |
| 5.G | Antibiotic resistance | 13 | 0.79 | 8 | 0.60 | -0.19 | 7 | 0.63 | -0.15 |
| 5.H | Conserved hypothetical proteins | 224 | 13.54 | 199 | 14.94 | 1.40 | 173 | 15.61 | 2.07 |
| 5.I | Unknown function | 138 | 8.34 | 113 | 8.48 | 0.14 | 96 | 8.66 | 0.32 |
| 6.A | Miscellaneous | 75 | 4.53 | 62 | 4.65 | 0.12 | 58 | 5.23 | 0.70 |
| sum |  | 1654 |  | 1332 |  |  | 1108 |  |  |

^1^Functional Categories were assigned by: Parkhill J, Wren BW, Mungall K, *et al*: **The genome sequence of the food-borne pathogen *Campylobacter jejuni* reveals hypervariable sequences**. *Nature* 2000, **403**(6770):665-668.

.
