# Supplementary material for: A proteome-wide protein interaction map for Campylobacter jejuni
Source: Genome Biol. 2007 Jul 5;8(7):R130. doi: 10.1186/gb-2007-8-7-r130 (PMC2323224; doi:10.1186/gb-2007-8-7-r130)
Supplement: Additional data file 3 — GO category representation amongst the proteins in CampyYTH v3.1 [file gb-2007-8-7-r130-S3.doc]

**Additional Data File 3.** Gene ontology (GO) category representation amongst the proteins in CampyYTH v3.1.

| **Function Description** | **Gene Ontology** | **Freq. in Proteome** | **Freq in Proteome (%)** | **Freq. in V3.1 Exp** | **Freq. in V3.1 Exp (%)** | **Enriched in V3.1 Exp (%)** | **Freq. in High Conf Set** | **Freq. in High Conf Set (%)** | **Enriched in High Conf Set (%)** |
| --- | --- | --- | --- | --- | --- | --- | --- | --- | --- |
| integral to membrane | Cellular Component | 127 | 7.68 | 75 | 5.63 | -2.05 | 43 | 3.88 | -3.80 |
| signal recognition particle (sensu Eukaryota) | Cellular Component | 3 | 0.18 | 1 | 0.08 | -0.11 | 1 | 0.09 | -0.09 |
| periplasmic space (sensu Proteobacteria) | Cellular Component | 11 | 0.67 | 8 | 0.60 | -0.06 | 4 | 0.36 | -0.30 |
| cell wall (sensu Bacteria) | Cellular Component | 1 | 0.06 | 0 | 0.00 | -0.06 | 0 | 0.00 | -0.06 |
| respiratory chain complex IV (sensu Bacteria) | Cellular Component | 1 | 0.06 | 0 | 0.00 | -0.06 | 0 | 0.00 | -0.06 |
| mitochondrial electron transport chain | Cellular Component | 1 | 0.06 | 0 | 0.00 | -0.06 | 0 | 0.00 | -0.06 |
| chromosome | Cellular Component | 2 | 0.12 | 1 | 0.08 | -0.05 | 1 | 0.09 | -0.03 |
| periplasmic space | Cellular Component | 3 | 0.18 | 2 | 0.15 | -0.03 | 1 | 0.09 | -0.09 |
| type II protein secretion system complex | Cellular Component | 3 | 0.18 | 2 | 0.15 | -0.03 | 2 | 0.18 | 0.00 |
| proton-transporting two-sector ATPase complex | Cellular Component | 11 | 0.67 | 9 | 0.68 | 0.01 | 7 | 0.63 | -0.03 |
| ribonucleoprotein complex | Cellular Component | 6 | 0.36 | 5 | 0.38 | 0.01 | 3 | 0.27 | -0.09 |
| outer membrane (sensu Proteobacteria) | Cellular Component | 6 | 0.36 | 5 | 0.38 | 0.01 | 4 | 0.36 | 0.00 |
| alpha DNA polymerase:primase complex | Cellular Component | 1 | 0.06 | 1 | 0.08 | 0.01 | 0 | 0.00 | -0.06 |
| DNA replication factor C complex | Cellular Component | 1 | 0.06 | 1 | 0.08 | 0.01 | 1 | 0.09 | 0.03 |
| phosphopyruvate hydratase complex | Cellular Component | 1 | 0.06 | 1 | 0.08 | 0.01 | 1 | 0.09 | 0.03 |
| imidazoleglycerol-phosphate synthase complex | Cellular Component | 1 | 0.06 | 1 | 0.08 | 0.01 | 1 | 0.09 | 0.03 |
| proteasome core complex (sensu Eukaryota) | Cellular Component | 1 | 0.06 | 1 | 0.08 | 0.01 | 1 | 0.09 | 0.03 |
| tricarboxylic acid cycle enzyme complex | Cellular Component | 1 | 0.06 | 1 | 0.08 | 0.01 | 1 | 0.09 | 0.03 |
| 3-isopropylmalate dehydratase complex | Cellular Component | 2 | 0.12 | 2 | 0.15 | 0.03 | 2 | 0.18 | 0.06 |
| ferredoxin hydrogenase complex | Cellular Component | 3 | 0.18 | 3 | 0.23 | 0.04 | 2 | 0.18 | 0.00 |
| ribosome | Cellular Component | 54 | 3.26 | 45 | 3.38 | 0.11 | 33 | 2.98 | -0.29 |
| intracellular | Cellular Component | 72 | 4.35 | 61 | 4.58 | 0.23 | 47 | 4.24 | -0.11 |
| transporter activity | Molecular Function | 55 | 3.33 | 31 | 2.33 | -1.00 | 19 | 1.71 | -1.61 |
| transferase activity | Molecular Function | 191 | 11.55 | 143 | 10.74 | -0.81 | 112 | 10.11 | -1.44 |
| DNA binding | Molecular Function | 69 | 4.17 | 47 | 3.53 | -0.64 | 36 | 3.25 | -0.92 |
| hydrolase activity | Molecular Function | 95 | 5.74 | 69 | 5.18 | -0.56 | 52 | 4.69 | -1.05 |
| magnesium ion binding | Molecular Function | 23 | 1.39 | 15 | 1.13 | -0.26 | 14 | 1.26 | -0.13 |
| protein binding | Molecular Function | 9 | 0.54 | 4 | 0.30 | -0.24 | 2 | 0.18 | -0.36 |
| two-component sensor activity | Molecular Function | 10 | 0.60 | 5 | 0.38 | -0.23 | 4 | 0.36 | -0.24 |
| methyltransferase activity | Molecular Function | 26 | 1.57 | 18 | 1.35 | -0.22 | 16 | 1.44 | -0.13 |
| kinase activity | Molecular Function | 32 | 1.93 | 23 | 1.73 | -0.21 | 20 | 1.81 | -0.13 |
| oxidoreductase activity | Molecular Function | 99 | 5.99 | 77 | 5.78 | -0.20 | 58 | 5.23 | -0.75 |
| signal transducer activity | Molecular Function | 18 | 1.09 | 12 | 0.90 | -0.19 | 11 | 0.99 | -0.10 |
| damaged DNA binding | Molecular Function | 4 | 0.24 | 1 | 0.08 | -0.17 | 1 | 0.09 | -0.15 |
| peptidase activity | Molecular Function | 15 | 0.91 | 10 | 0.75 | -0.16 | 4 | 0.36 | -0.55 |
| binding | Molecular Function | 10 | 0.60 | 6 | 0.45 | -0.15 | 3 | 0.27 | -0.33 |
| ATPase activity, coupled to transmembrane movement of substances | Molecular Function | 6 | 0.36 | 3 | 0.23 | -0.14 | 3 | 0.27 | -0.09 |
| ligase activity | Molecular Function | 58 | 3.51 | 45 | 3.38 | -0.13 | 38 | 3.43 | -0.08 |
| transaminase activity | Molecular Function | 17 | 1.03 | 12 | 0.90 | -0.13 | 12 | 1.08 | 0.06 |
| unfolded protein binding | Molecular Function | 12 | 0.73 | 8 | 0.60 | -0.12 | 6 | 0.54 | -0.18 |
| nuclease activity | Molecular Function | 12 | 0.73 | 8 | 0.60 | -0.12 | 7 | 0.63 | -0.09 |
| hydrolase activity, acting on acid anhydrides, catalyzing transmembrane movement of substances | Molecular Function | 7 | 0.42 | 4 | 0.30 | -0.12 | 2 | 0.18 | -0.24 |
| tetracycline:hydrogen antiporter activity | Molecular Function | 2 | 0.12 | 0 | 0.00 | -0.12 | 0 | 0.00 | -0.12 |
| ATPase activity, coupled to transmembrane movement of ions, phosphorylative mechanism | Molecular Function | 2 | 0.12 | 0 | 0.00 | -0.12 | 0 | 0.00 | -0.12 |
| helicase activity | Molecular Function | 13 | 0.79 | 9 | 0.68 | -0.11 | 7 | 0.63 | -0.15 |
| metal ion binding | Molecular Function | 8 | 0.48 | 5 | 0.38 | -0.11 | 4 | 0.36 | -0.12 |
| transferase activity, transferring phosphorus-containing groups | Molecular Function | 8 | 0.48 | 5 | 0.38 | -0.11 | 4 | 0.36 | -0.12 |
| metal ion transporter activity | Molecular Function | 3 | 0.18 | 1 | 0.08 | -0.11 | 1 | 0.09 | -0.09 |
| translation initiation factor activity | Molecular Function | 3 | 0.18 | 1 | 0.08 | -0.11 | 0 | 0.00 | -0.18 |
| hydrolase activity, acting on carbon-nitrogen (but not peptide) bonds | Molecular Function | 3 | 0.18 | 1 | 0.08 | -0.11 | 1 | 0.09 | -0.09 |
| peptidyl-prolyl cis-trans isomerase activity | Molecular Function | 3 | 0.18 | 1 | 0.08 | -0.11 | 1 | 0.09 | -0.09 |
| transferase activity, transferring glycosyl groups | Molecular Function | 14 | 0.85 | 10 | 0.75 | -0.10 | 8 | 0.72 | -0.12 |
| pseudouridine synthase activity | Molecular Function | 4 | 0.24 | 2 | 0.15 | -0.09 | 1 | 0.09 | -0.15 |
| protein translocase activity | Molecular Function | 6 | 0.36 | 4 | 0.30 | -0.06 | 3 | 0.27 | -0.09 |
| metallopeptidase activity | Molecular Function | 6 | 0.36 | 4 | 0.30 | -0.06 | 3 | 0.27 | -0.09 |
| porin activity | Molecular Function | 1 | 0.06 | 0 | 0.00 | -0.06 | 0 | 0.00 | -0.06 |
| sugar porter activity | Molecular Function | 1 | 0.06 | 0 | 0.00 | -0.06 | 0 | 0.00 | -0.06 |
| hydrolase activity, acting on carbon-nitrogen (but not peptide) bonds, in cyclic amides | Molecular Function | 1 | 0.06 | 0 | 0.00 | -0.06 | 0 | 0.00 | -0.06 |
| hydrolase activity, acting on carbon-sulfur bonds | Molecular Function | 1 | 0.06 | 0 | 0.00 | -0.06 | 0 | 0.00 | -0.06 |
| hydrolase activity, acting on ester bonds | Molecular Function | 1 | 0.06 | 0 | 0.00 | -0.06 | 0 | 0.00 | -0.06 |
| UDP-3-O-[3-hydroxymyristoyl] N-acetylglucosamine deacetylase activity | Molecular Function | 1 | 0.06 | 0 | 0.00 | -0.06 | 0 | 0.00 | -0.06 |
| DNA topoisomerase type I activity | Molecular Function | 1 | 0.06 | 0 | 0.00 | -0.06 | 0 | 0.00 | -0.06 |
| adenylosuccinate synthase activity | Molecular Function | 1 | 0.06 | 0 | 0.00 | -0.06 | 0 | 0.00 | -0.06 |
| carbon-nitrogen ligase activity, with glutamine as amido-N-donor | Molecular Function | 1 | 0.06 | 0 | 0.00 | -0.06 | 0 | 0.00 | -0.06 |
| 2-C-methyl-D-erythritol 2,4-cyclodiphosphate synthase activity | Molecular Function | 1 | 0.06 | 0 | 0.00 | -0.06 | 0 | 0.00 | -0.06 |
| oxo-acid-lyase activity | Molecular Function | 1 | 0.06 | 0 | 0.00 | -0.06 | 0 | 0.00 | -0.06 |
| cysteine synthase activity | Molecular Function | 1 | 0.06 | 0 | 0.00 | -0.06 | 0 | 0.00 | -0.06 |
| dihydroorotate dehydrogenase activity | Molecular Function | 1 | 0.06 | 0 | 0.00 | -0.06 | 0 | 0.00 | -0.06 |
| proline dehydrogenase activity | Molecular Function | 1 | 0.06 | 0 | 0.00 | -0.06 | 0 | 0.00 | -0.06 |
| glutamate synthase activity | Molecular Function | 1 | 0.06 | 0 | 0.00 | -0.06 | 0 | 0.00 | -0.06 |
| transferase activity, transferring acyl groups, acyl groups converted into alkyl on transfer | Molecular Function | 1 | 0.06 | 0 | 0.00 | -0.06 | 0 | 0.00 | -0.06 |
| 3-deoxy-7-phosphoheptulonate synthase activity | Molecular Function | 1 | 0.06 | 0 | 0.00 | -0.06 | 0 | 0.00 | -0.06 |
| nucleotidyltransferase activity | Molecular Function | 22 | 1.33 | 17 | 1.28 | -0.05 | 15 | 1.35 | 0.02 |
| structural molecule activity | Molecular Function | 12 | 0.73 | 9 | 0.68 | -0.05 | 6 | 0.54 | -0.18 |
| translation elongation factor activity | Molecular Function | 7 | 0.42 | 5 | 0.38 | -0.05 | 4 | 0.36 | -0.06 |
| oxygen transporter activity | Molecular Function | 2 | 0.12 | 1 | 0.08 | -0.05 | 1 | 0.09 | -0.03 |
| amino acid-polyamine transporter activity | Molecular Function | 2 | 0.12 | 1 | 0.08 | -0.05 | 1 | 0.09 | -0.03 |
| sulfuric ester hydrolase activity | Molecular Function | 2 | 0.12 | 1 | 0.08 | -0.05 | 1 | 0.09 | -0.03 |
| serine-type peptidase activity | Molecular Function | 2 | 0.12 | 1 | 0.08 | -0.05 | 1 | 0.09 | -0.03 |
| biotin carboxylase activity | Molecular Function | 2 | 0.12 | 1 | 0.08 | -0.05 | 1 | 0.09 | -0.03 |
| carbamoyl-phosphate synthase activity | Molecular Function | 2 | 0.12 | 1 | 0.08 | -0.05 | 1 | 0.09 | -0.03 |
| acetolactate synthase activity | Molecular Function | 2 | 0.12 | 1 | 0.08 | -0.05 | 1 | 0.09 | -0.03 |
| isomerase activity | Molecular Function | 28 | 1.69 | 22 | 1.65 | -0.04 | 18 | 1.62 | -0.07 |
| receptor activity | Molecular Function | 3 | 0.18 | 2 | 0.15 | -0.03 | 0 | 0.00 | -0.18 |
| neurotransmitter:sodium symporter activity | Molecular Function | 3 | 0.18 | 2 | 0.15 | -0.03 | 0 | 0.00 | -0.18 |
| DNA topoisomerase activity | Molecular Function | 3 | 0.18 | 2 | 0.15 | -0.03 | 1 | 0.09 | -0.09 |
| sodium:dicarboxylate symporter activity | Molecular Function | 4 | 0.24 | 3 | 0.23 | -0.02 | 2 | 0.18 | -0.06 |
| disulfide oxidoreductase activity | Molecular Function | 4 | 0.24 | 3 | 0.23 | -0.02 | 3 | 0.27 | 0.03 |
| cytochrome-c oxidase activity | Molecular Function | 4 | 0.24 | 3 | 0.23 | -0.02 | 3 | 0.27 | 0.03 |
| succinate dehydrogenase activity | Molecular Function | 4 | 0.24 | 3 | 0.23 | -0.02 | 2 | 0.18 | -0.06 |
| lyase activity | Molecular Function | 61 | 3.69 | 49 | 3.68 | -0.01 | 43 | 3.88 | 0.19 |
| protein transporter activity | Molecular Function | 15 | 0.91 | 12 | 0.90 | -0.01 | 9 | 0.81 | -0.09 |
| pyridoxal phosphate binding | Molecular Function | 5 | 0.30 | 4 | 0.30 | 0.00 | 4 | 0.36 | 0.06 |
| hydro-lyase activity | Molecular Function | 5 | 0.30 | 4 | 0.30 | 0.00 | 3 | 0.27 | -0.03 |
| sugar binding | Molecular Function | 6 | 0.36 | 5 | 0.38 | 0.01 | 4 | 0.36 | 0.00 |
| transcriptional activator activity | Molecular Function | 1 | 0.06 | 1 | 0.08 | 0.01 | 1 | 0.09 | 0.03 |
| sodium:amino acid symporter activity | Molecular Function | 1 | 0.06 | 1 | 0.08 | 0.01 | 1 | 0.09 | 0.03 |
| carrier activity | Molecular Function | 1 | 0.06 | 1 | 0.08 | 0.01 | 1 | 0.09 | 0.03 |
| thioredoxin peroxidase activity | Molecular Function | 1 | 0.06 | 1 | 0.08 | 0.01 | 1 | 0.09 | 0.03 |
| thioredoxin-disulfide reductase activity | Molecular Function | 1 | 0.06 | 1 | 0.08 | 0.01 | 1 | 0.09 | 0.03 |
| FAD binding | Molecular Function | 1 | 0.06 | 1 | 0.08 | 0.01 | 1 | 0.09 | 0.03 |
| FMN binding | Molecular Function | 1 | 0.06 | 1 | 0.08 | 0.01 | 1 | 0.09 | 0.03 |
| chaperone binding | Molecular Function | 1 | 0.06 | 1 | 0.08 | 0.01 | 1 | 0.09 | 0.03 |
| protein homodimerization activity | Molecular Function | 1 | 0.06 | 1 | 0.08 | 0.01 | 1 | 0.09 | 0.03 |
| biotin binding | Molecular Function | 1 | 0.06 | 1 | 0.08 | 0.01 | 0 | 0.00 | -0.06 |
| carbohydrate binding | Molecular Function | 1 | 0.06 | 1 | 0.08 | 0.01 | 0 | 0.00 | -0.06 |
| 3,4-dihydroxy-2-butanone-4-phosphate synthase activity | Molecular Function | 1 | 0.06 | 1 | 0.08 | 0.01 | 1 | 0.09 | 0.03 |
| phosphoric ester hydrolase activity | Molecular Function | 1 | 0.06 | 1 | 0.08 | 0.01 | 1 | 0.09 | 0.03 |
| cysteine-type peptidase activity | Molecular Function | 1 | 0.06 | 1 | 0.08 | 0.01 | 1 | 0.09 | 0.03 |
| endopeptidase activity | Molecular Function | 1 | 0.06 | 1 | 0.08 | 0.01 | 1 | 0.09 | 0.03 |
| intramolecular oxidoreductase activity, interconverting keto- and enol-groups | Molecular Function | 1 | 0.06 | 1 | 0.08 | 0.01 | 1 | 0.09 | 0.03 |
| UDP-galactopyranose mutase activity | Molecular Function | 1 | 0.06 | 1 | 0.08 | 0.01 | 0 | 0.00 | -0.06 |
| chorismate mutase activity | Molecular Function | 1 | 0.06 | 1 | 0.08 | 0.01 | 1 | 0.09 | 0.03 |
| acid-amino acid ligase activity | Molecular Function | 1 | 0.06 | 1 | 0.08 | 0.01 | 1 | 0.09 | 0.03 |
| phosphoribosylamine-glycine ligase activity | Molecular Function | 1 | 0.06 | 1 | 0.08 | 0.01 | 1 | 0.09 | 0.03 |
| argininosuccinate synthase activity | Molecular Function | 1 | 0.06 | 1 | 0.08 | 0.01 | 1 | 0.09 | 0.03 |
| CTP synthase activity | Molecular Function | 1 | 0.06 | 1 | 0.08 | 0.01 | 1 | 0.09 | 0.03 |
| ferrochelatase activity | Molecular Function | 1 | 0.06 | 1 | 0.08 | 0.01 | 1 | 0.09 | 0.03 |
| carbon-carbon lyase activity | Molecular Function | 1 | 0.06 | 1 | 0.08 | 0.01 | 0 | 0.00 | -0.06 |
| 1-aminocyclopropane-1-carboxylate synthase activity | Molecular Function | 1 | 0.06 | 1 | 0.08 | 0.01 | 1 | 0.09 | 0.03 |
| oxidoreductase activity, acting on CH-OH group of donors | Molecular Function | 1 | 0.06 | 1 | 0.08 | 0.01 | 1 | 0.09 | 0.03 |
| oxidoreductase activity, acting on CH2 groups, disulfide as acceptor | Molecular Function | 1 | 0.06 | 1 | 0.08 | 0.01 | 0 | 0.00 | -0.06 |
| oxidoreductase activity, acting on CH2 groups, NAD or NADP as acceptor | Molecular Function | 1 | 0.06 | 1 | 0.08 | 0.01 | 1 | 0.09 | 0.03 |
| nitrate reductase activity | Molecular Function | 1 | 0.06 | 1 | 0.08 | 0.01 | 0 | 0.00 | -0.06 |
| oxidoreductase activity, acting on sulfur group of donors, NAD or NADP as acceptor | Molecular Function | 1 | 0.06 | 1 | 0.08 | 0.01 | 0 | 0.00 | -0.06 |
| superoxide dismutase activity | Molecular Function | 1 | 0.06 | 1 | 0.08 | 0.01 | 0 | 0.00 | -0.06 |
| oxidoreductase activity, acting on the CH-CH group of donors | Molecular Function | 1 | 0.06 | 1 | 0.08 | 0.01 | 1 | 0.09 | 0.03 |
| oxidoreductase activity, acting on the CH-NH2 group of donors, NAD or NADP as acceptor | Molecular Function | 1 | 0.06 | 1 | 0.08 | 0.01 | 1 | 0.09 | 0.03 |
| transferase activity, transferring amino-acyl groups | Molecular Function | 1 | 0.06 | 1 | 0.08 | 0.01 | 1 | 0.09 | 0.03 |
| transferase activity, transferring groups other than amino-acyl groups | Molecular Function | 1 | 0.06 | 1 | 0.08 | 0.01 | 1 | 0.09 | 0.03 |
| 1-deoxy-D-xylulose-5-phosphate synthase activity | Molecular Function | 1 | 0.06 | 1 | 0.08 | 0.01 | 1 | 0.09 | 0.03 |
| transaldolase activity | Molecular Function | 1 | 0.06 | 1 | 0.08 | 0.01 | 1 | 0.09 | 0.03 |
| transketolase activity | Molecular Function | 1 | 0.06 | 1 | 0.08 | 0.01 | 1 | 0.09 | 0.03 |
| dihydropteroate synthase activity | Molecular Function | 1 | 0.06 | 1 | 0.08 | 0.01 | 0 | 0.00 | -0.06 |
| 3-deoxy-8-phosphooctulonate synthase activity | Molecular Function | 1 | 0.06 | 1 | 0.08 | 0.01 | 1 | 0.09 | 0.03 |
| hydroxymethylbilane synthase activity | Molecular Function | 1 | 0.06 | 1 | 0.08 | 0.01 | 0 | 0.00 | -0.06 |
| methionine adenosyltransferase activity | Molecular Function | 1 | 0.06 | 1 | 0.08 | 0.01 | 1 | 0.09 | 0.03 |
| 3-phosphoshikimate 1-carboxyvinyltransferase activity | Molecular Function | 1 | 0.06 | 1 | 0.08 | 0.01 | 1 | 0.09 | 0.03 |
| tRNA isopentenyltransferase activity | Molecular Function | 1 | 0.06 | 1 | 0.08 | 0.01 | 1 | 0.09 | 0.03 |
| UDP-N-acetylglucosamine 1-carboxyvinyltransferase activity | Molecular Function | 1 | 0.06 | 1 | 0.08 | 0.01 | 0 | 0.00 | -0.06 |
| transferase activity, transferring hexosyl groups | Molecular Function | 1 | 0.06 | 1 | 0.08 | 0.01 | 0 | 0.00 | -0.06 |
| phosphotransferase activity, carboxyl group as acceptor | Molecular Function | 1 | 0.06 | 1 | 0.08 | 0.01 | 1 | 0.09 | 0.03 |
| adenylate cyclase activity | Molecular Function | 1 | 0.06 | 1 | 0.08 | 0.01 | 0 | 0.00 | -0.06 |
| dCTP deaminase activity | Molecular Function | 1 | 0.06 | 1 | 0.08 | 0.01 | 1 | 0.09 | 0.03 |
| cytochrome-c peroxidase activity | Molecular Function | 2 | 0.12 | 2 | 0.15 | 0.03 | 1 | 0.09 | -0.03 |
| ice binding | Molecular Function | 2 | 0.12 | 2 | 0.15 | 0.03 | 2 | 0.18 | 0.06 |
| hydrolase activity, acting on acid anhydrides, in phosphorus-containing anhydrides | Molecular Function | 2 | 0.12 | 2 | 0.15 | 0.03 | 2 | 0.18 | 0.06 |
| racemase and epimerase activity, acting on amino acids and derivatives | Molecular Function | 2 | 0.12 | 2 | 0.15 | 0.03 | 2 | 0.18 | 0.06 |
| DNA topoisomerase (ATP-hydrolyzing) activity | Molecular Function | 2 | 0.12 | 2 | 0.15 | 0.03 | 1 | 0.09 | -0.03 |
| intramolecular transferase activity, phosphotransferases | Molecular Function | 2 | 0.12 | 2 | 0.15 | 0.03 | 2 | 0.18 | 0.06 |
| pyruvate carboxylase activity | Molecular Function | 2 | 0.12 | 2 | 0.15 | 0.03 | 1 | 0.09 | -0.03 |
| DNA-(apurinic or apyrimidinic site) lyase activity | Molecular Function | 2 | 0.12 | 2 | 0.15 | 0.03 | 2 | 0.18 | 0.06 |
| transferase activity, transferring alkyl or aryl (other than methyl) groups | Molecular Function | 2 | 0.12 | 2 | 0.15 | 0.03 | 1 | 0.09 | -0.03 |
| riboflavin synthase activity | Molecular Function | 2 | 0.12 | 2 | 0.15 | 0.03 | 1 | 0.09 | -0.03 |
| thiamin-phosphate diphosphorylase activity | Molecular Function | 2 | 0.12 | 2 | 0.15 | 0.03 | 2 | 0.18 | 0.06 |
| transferase activity, transferring pentosyl groups | Molecular Function | 2 | 0.12 | 2 | 0.15 | 0.03 | 2 | 0.18 | 0.06 |
| carboxyl- and carbamoyltransferase activity | Molecular Function | 2 | 0.12 | 2 | 0.15 | 0.03 | 2 | 0.18 | 0.06 |
| peroxidase activity | Molecular Function | 3 | 0.18 | 3 | 0.23 | 0.04 | 2 | 0.18 | 0.00 |
| protein disulfide oxidoreductase activity | Molecular Function | 3 | 0.18 | 3 | 0.23 | 0.04 | 3 | 0.27 | 0.09 |
| oxidoreductase activity, acting on NADH or NADPH | Molecular Function | 3 | 0.18 | 3 | 0.23 | 0.04 | 1 | 0.09 | -0.09 |
| hydrolase activity, acting on glycosyl bonds | Molecular Function | 4 | 0.24 | 4 | 0.30 | 0.06 | 3 | 0.27 | 0.03 |
| oxidoreductase activity, acting on the CH-OH group of donors, NAD or NADP as acceptor | Molecular Function | 4 | 0.24 | 4 | 0.30 | 0.06 | 3 | 0.27 | 0.03 |
| catalytic activity | Molecular Function | 97 | 5.86 | 79 | 5.93 | 0.07 | 67 | 6.05 | 0.18 |
| structural constituent of ribosome | Molecular Function | 54 | 3.26 | 45 | 3.38 | 0.11 | 33 | 2.98 | -0.29 |
| carboxy-lyase activity | Molecular Function | 9 | 0.54 | 9 | 0.68 | 0.13 | 7 | 0.63 | 0.09 |
| electron transporter activity | Molecular Function | 29 | 1.75 | 26 | 1.95 | 0.20 | 18 | 1.62 | -0.13 |
| transport | Biological Process | 93 | 5.62 | 57 | 4.28 | -1.34 | 37 | 3.34 | -2.28 |
| two-component signal transduction system (phosphorelay) | Biological Process | 16 | 0.97 | 8 | 0.60 | -0.37 | 8 | 0.72 | -0.25 |
| signal transduction | Biological Process | 22 | 1.33 | 14 | 1.05 | -0.28 | 12 | 1.08 | -0.25 |
| protein transport | Biological Process | 18 | 1.09 | 11 | 0.83 | -0.26 | 8 | 0.72 | -0.37 |
| lipid biosynthesis | Biological Process | 9 | 0.54 | 4 | 0.30 | -0.24 | 4 | 0.36 | -0.18 |
| cytokinesis | Biological Process | 13 | 0.79 | 8 | 0.60 | -0.19 | 5 | 0.45 | -0.33 |
| cell cycle | Biological Process | 4 | 0.24 | 2 | 0.15 | -0.09 | 1 | 0.09 | -0.15 |
| pathogenesis | Biological Process | 4 | 0.24 | 2 | 0.15 | -0.09 | 2 | 0.18 | -0.06 |
| ciliary or flagellar motility | Biological Process | 21 | 1.27 | 16 | 1.20 | -0.07 | 13 | 1.17 | -0.10 |
| cell adhesion | Biological Process | 1 | 0.06 | 0 | 0.00 | -0.06 | 0 | 0.00 | -0.06 |
| biosynthesis | Biological Process | 37 | 2.24 | 29 | 2.18 | -0.06 | 24 | 2.17 | -0.07 |
| DNA repair | Biological Process | 22 | 1.33 | 17 | 1.28 | -0.05 | 14 | 1.26 | -0.07 |
| response to unfolded protein | Biological Process | 7 | 0.42 | 5 | 0.38 | -0.05 | 3 | 0.27 | -0.15 |
| chromosome segregation | Biological Process | 2 | 0.12 | 1 | 0.08 | -0.05 | 1 | 0.09 | -0.03 |
| neurotransmitter transport | Biological Process | 3 | 0.18 | 2 | 0.15 | -0.03 | 0 | 0.00 | -0.18 |
| protein metabolism | Biological Process | 4 | 0.24 | 3 | 0.23 | -0.02 | 2 | 0.18 | -0.06 |
| electron transport | Biological Process | 72 | 4.35 | 58 | 4.35 | 0.00 | 45 | 4.06 | -0.29 |
| membrane organization and biogenesis | Biological Process | 1 | 0.06 | 1 | 0.08 | 0.01 | 0 | 0.00 | -0.06 |
| peptide transport | Biological Process | 1 | 0.06 | 1 | 0.08 | 0.01 | 1 | 0.09 | 0.03 |
| cellular morphogenesis | Biological Process | 1 | 0.06 | 1 | 0.08 | 0.01 | 1 | 0.09 | 0.03 |
| DNA mediated transformation | Biological Process | 1 | 0.06 | 1 | 0.08 | 0.01 | 1 | 0.09 | 0.03 |
| nitrogen fixation | Biological Process | 1 | 0.06 | 1 | 0.08 | 0.01 | 1 | 0.09 | 0.03 |
| chromatin silencing | Biological Process | 1 | 0.06 | 1 | 0.08 | 0.01 | 1 | 0.09 | 0.03 |
| metabolism | Biological Process | 73 | 4.41 | 59 | 4.43 | 0.02 | 52 | 4.69 | 0.28 |
| chemotaxis | Biological Process | 22 | 1.33 | 18 | 1.35 | 0.02 | 16 | 1.44 | 0.11 |
| response to freezing | Biological Process | 2 | 0.12 | 2 | 0.15 | 0.03 | 2 | 0.18 | 0.06 |
| sulfur metabolism | Biological Process | 2 | 0.12 | 2 | 0.15 | 0.03 | 2 | 0.18 | 0.06 |
| aromatic compound metabolism | Biological Process | 2 | 0.12 | 2 | 0.15 | 0.03 | 2 | 0.18 | 0.06 |
| homoiothermy | Biological Process | 2 | 0.12 | 2 | 0.15 | 0.03 | 2 | 0.18 | 0.06 |
| response to DNA damage stimulus | Biological Process | 3 | 0.18 | 3 | 0.23 | 0.04 | 2 | 0.18 | 0.00 |
| one-carbon compound metabolism | Biological Process | 3 | 0.18 | 3 | 0.23 | 0.04 | 3 | 0.27 | 0.09 |
