# Supplementary material for: A proteome-wide protein interaction map for Campylobacter jejuni
Source: Genome Biol. 2007 Jul 5;8(7):R130. doi: 10.1186/gb-2007-8-7-r130 (PMC2323224; doi:10.1186/gb-2007-8-7-r130)
Supplement: Additional data file 4 — C. jejuni genes that were toxic or inhibitory to yeast growth [file gb-2007-8-7-r130-S4.doc]

**Additional Data File 4.** List of *C. jejuni* genes whose expression was toxic or inhibitory to yeast growth.

|  | **Gene** | **Growth** | **Growth** | **Level** | **Interactions** | **Interactions** |
| --- | --- | --- | --- | --- | --- | --- |
| **Gene ID** | **Symbol ID** | **on WD^1^** | **on WGR^2^** | **of Toxicity** | **as AD^3^** | **(HC) as AD^4^** |
| Cj0018c | Cj0018c | 1 | 0 | high | 6 | 3 |
| Cj0260c | Cj0260c | 1 | 0 | high | 9 | 2 |
| Cj0239c | Cj0239c | 3 | 1 | medium | 5 | 5 |
| Cj0663c | hslV | 3 | 1 | medium | 7 | 1 |
| Cj0725c | mog | 3 | 1 | medium | 2 | 2 |
| Cj1220 | groES | 3 | 1 | medium | 4 | 4 |
| Cj0015c | Cj0015c | 3 | 1 | medium | 3 | 3 |
| Cj0026c | Cj0026c | 3 | 1 | medium | 3 | 3 |
| Cj0040 | Cj0040 | 3 | 1 | medium | 1 | 0 |
| Cj0066c | aroQ | 3 | 1 | medium | 13 | 3 |
| Cj0101 | Cj0101 | 3 | 1 | medium | 4 | 4 |
| Cj0163c | Cj0163c | 3 | 1 | medium | 12 | 2 |
| Cj0194 | folE | 3 | 1 | medium | 1 | 1 |
| Cj0233c | pyrE | 3 | 1 | medium | 17 | 3 |
| Cj0237 | cynT | 3 | 1 | medium | 5 | 2 |
| Cj0252 | moaC | 3 | 1 | medium | 12 | 3 |
| Cj0383c | ribH | 3 | 1 | medium | 21 | 6 |
| Cj0488 | Cj0488 | 3 | 1 | medium | 4 | 4 |
| Cj0510c | Cj0510c | 3 | 1 | medium | 4 | 4 |
| Cj0513 | Cj0513 | 3 | 1 | medium | 2 | 2 |
| Cj0539 | Cj0539 | 3 | 1 | medium | 2 | 2 |
| Cj0547 | flaG | 3 | 1 | medium | 2 | 2 |
| Cj0702 | purE | 3 | 1 | medium | 3 | 1 |
| Cj0758 | grpE | 3 | 1 | medium | 2 | 1 |
| Cj0797c | Cj0797c | 3 | 1 | medium | 85 | 75 |
| Cj0925 | rpiB | 3 | 1 | medium | 3 | 2 |
| Cj0945c | Cj0945c | 3 | 1 | medium | 0 | 0 |
| Cj1071 | ssb | 3 | 1 | medium | 4 | 4 |
| Cj1086c | Cj1086c | 3 | 1 | medium | 2 | 1 |
| Cj1123c | wlaI | 3 | 1 | medium | 3 | 1 |
| Cj1149c | gmhA | 3 | 1 | medium | 1 | 1 |
| Cj1156 | rho | 3 | 1 | medium | 2 | 2 |
| Cj1255 | Cj1255 | 3 | 1 | medium | 6 | 5 |
| Cj1269c | amiA | 3 | 1 | medium | 1 | 0 |
| Cj1284 | ktrA | 3 | 1 | medium | 1 | 1 |
| Cj1388 | Cj1388 | 3 | 1 | medium | 2 | 2 |
| Cj1410c | Cj1410c | 3 | 1 | medium | 0 | 0 |
| Cj1424c | gmhA2 | 3 | 1 | medium | 3 | 3 |
| Cj1534c | Cj1534c | 3 | 1 | medium | 25 | 3 |
| Cj1724c | Cj1724c | 3 | 1 | medium | 3 | 3 |
| Cj0146c | trxB | 3 | 2 | low | 3 | 3 |
| Cj0284c | cheA | 3 | 2 | low | 11 | 4 |
| Cj0857c | moeA | 3 | 2 | low | 4 | 2 |
| Cj0891c | serA | 3 | 2 | low | 4 | 3 |
| Cj1272c | spoT | 3 | 2 | low | 21 | 12 |
| Cj1362 | ruvB | 3 | 2 | low | 4 | 4 |
| Cj0012c | Cj0012c | 3 | 2 | low | 0 | 0 |
| Cj0027 | pyrG | 3 | 2 | low | 3 | 3 |
| Cj0036 | Cj0036 | 3 | 2 | low | 0 | 0 |
| Cj0039c | typA | 3 | 2 | low | 5 | 1 |
| Cj0054c | Cj0054c | 3 | 2 | low | 10 | 4 |
| Cj0064c | flhF | 3 | 2 | low | 6 | 5 |
| Cj0097 | proB | 3 | 2 | low | 3 | 3 |
| Cj0172c | Cj0172c | 3 | 2 | low | 14 | 3 |
| Cj0229 | Cj0229 | 3 | 2 | low | 11 | 1 |
| Cj0257 | dgkA | 3 | 2 | low | 2 | 2 |
| Cj0258 | Cj0258 | 3 | 2 | low | 4 | 1 |
| Cj0316 | pheA | 3 | 2 | low | 3 | 3 |
| Cj0327 | Cj0327 | 3 | 2 | low | 6 | 3 |
| Cj0360 | Cj0360 | 3 | 2 | low | 2 | 1 |
| Cj0364 | Cj0364 | 3 | 2 | low | 3 | 1 |
| Cj0400 | fur | 3 | 2 | low | 15 | 3 |
| Cj0422c | Cj0422c | 3 | 2 | low | 5 | 5 |
| Cj0442 | fabF | 3 | 2 | low | 37 | 3 |
| Cj0447 | Cj0447 | 3 | 2 | low | 20 | 4 |
| Cj0448c | Cj0448c | 3 | 2 | low | 4 | 3 |
| Cj0459c | Cj0459c | 3 | 2 | low | 40 | 4 |
| Cj0477 | rplL | 3 | 2 | low | 27 | 4 |
| Cj0511 | Cj0511 | 3 | 2 | low | 0 | 0 |
| Cj0518 | htpG | 3 | 2 | low | 6 | 1 |
| Cj0548 | fliD | 3 | 2 | low | 4 | 1 |
| Cj0632 | ilvC | 3 | 2 | low | 3 | 3 |
| Cj0640c | aspS | 3 | 2 | low | 6 | 1 |
| Cj0665c | argG | 3 | 2 | low | 12 | 4 |
| Cj0703 | Cj0703 | 3 | 2 | low | 3 | 2 |
| Cj0764c | speA | 3 | 2 | low | 3 | 3 |
| Cj0779 | tpx | 3 | 2 | low | 1 | 1 |
| Cj0788 | Cj0788 | 3 | 2 | low | 10 | 4 |
| Cj0880c | Cj0880c | 3 | 2 | low | 3 | 2 |
| Cj0898 | Cj0898 | 3 | 2 | low | 3 | 2 |
| Cj0899c | thiJ | 3 | 2 | low | 1 | 1 |
| Cj0927 | apt | 3 | 2 | low | 5 | 1 |
| Cj0931c | argH | 3 | 2 | low | 4 | 3 |
| Cj0955c | purL | 3 | 2 | low | 1 | 0 |
| Cj0962 | Cj0962 | 3 | 2 | low | 10 | 5 |
| Cj0977 | Cj0977 | 3 | 2 | low | 9 | 2 |
| Cj1057c | Cj1057c | 3 | 2 | low | 3 | 3 |
| Cj1067 | pgsA | 3 | 2 | low | 1 | 0 |
| Cj1099 | Cj1099 | 3 | 2 | low | 5 | 3 |
| Cj1118c | cheY | 3 | 2 | low | 0 | 0 |
| Cj1205c | radA | 3 | 2 | low | 0 | 0 |
| Cj1221 | groEL | 3 | 2 | low | 2 | 0 |
| Cj1227c | Cj1227c | 3 | 2 | low | 0 | 0 |
| Cj1253 | pnp | 3 | 2 | low | 0 | 0 |
| Cj1316c | Cj1316c | 3 | 2 | low | 5 | 3 |
| Cj1347c | cdsA | 3 | 2 | low | 0 | 0 |
| Cj1357c | Cj1357c | 3 | 2 | low | 0 | 0 |
| Cj1364c | fumC | 3 | 2 | low | 3 | 1 |
| Cj1366c | glmS | 3 | 2 | low | 3 | 2 |
| Cj1378 | selA | 3 | 2 | low | 10 | 4 |
| Cj1385 | katA | 3 | 2 | low | 7 | 2 |
| Cj1402c | pgk | 3 | 2 | low | 4 | 4 |
| Cj1403c | gapA | 3 | 2 | low | 4 | 3 |
| Cj1405 | Cj1405 | 3 | 2 | low | 3 | 2 |
| Cj1443c | kpsF | 3 | 2 | low | 1 | 1 |
| Cj1519 | moeA2 | 3 | 2 | low | 3 | 3 |
| Cj1536c | galU | 3 | 2 | low | 1 | 1 |
| Cj1537c | acs | 3 | 2 | low | 4 | 3 |
| Cj1546 | Cj1546 | 3 | 2 | low | 0 | 0 |
| Cj1575c | Cj1575c | 3 | 2 | low | 2 | 2 |
| Cj1597 | hisG | 3 | 2 | low | 1 | 1 |
| Cj1599 | hisB | 3 | 2 | low | 2 | 2 |
| Cj1605c | dapD | 3 | 2 | low | 2 | 1 |
| Cj1673c | recA | 3 | 2 | low | 2 | 2 |

^1^Yeast were grown in medium lacking tryptophan (W) to select for the yeast two-hybrid vector expressing the *C. jejuni*-AD fusion proteins. The medium was supplemented with glucose (D), which represses expression from the inducible *GAL1* promoter and also serves as carbon source. The numbers correspond to yeast growth, which can range from 0 (no growth) to 3 (maximum growth). The scores represent growth under non-inducing conditions.

^2^Yeast were grown in medium lacking tryptophan (W) and supplemented with galactose (G) and raffinose. Galactose induces expression of the *GAL1* promoter, therefore the growth scores represent yeast growth when *C. jejuni* fusion proteins are expressed. Toxic or inhibitory proteins result in a lower growth score under inducing conditions than non-inducing conditions. The remainder of the *C. jejuni* AD fusion proteins not shown on this table had equal growth on glucose and on galactose-containing media.

^3^The number of protein interactions that include the protein AD fusion from the CampyYTH v3.1 set of 12,012 interactions.

^4^The number of higher confidence (HC) protein interactions that include the protein AD fusion.
