# Supplementary material for: A proteome-wide protein interaction map for Campylobacter jejuni
Source: Genome Biol. 2007 Jul 5;8(7):R130. doi: 10.1186/gb-2007-8-7-r130 (PMC2323224; doi:10.1186/gb-2007-8-7-r130)
Supplement: Additional data file 5 — Comparison of network features across organisms [file gb-2007-8-7-r130-S5.doc]

**Additional Data File 5. Comparison of network features across organisms^1^.**

| **Interaction datasets** | **Nodes** | **Edges** | **Diameter** | **Average pathlength** | **Degree exponent** | **Average clustering coefficient** | **Interactions**  **/ Protein** | **Average # interactions** | **Connected pairs^2^** | **Coverage**  **%** | **Data**  **source** |
| --- | --- | --- | --- | --- | --- | --- | --- | --- | --- | --- | --- |
| *Campylobacter jejuni* (All)^3^ | 1,332 | 12,012 | 6 | 2.9 | 1.28 | 0.10 | 9.0 | 17.5 | 99% | 80.5 | This study |
| *Campylobacter jejuni* (High confidence) | 1,108 | 3,209 | 9 | 3.8 | 1.53 | 0.05 | 2.9 | 5.4 | 95% | 67 | This study |
| *Helicobacter pylori* 26695 | 732 | 1,465 | 9 | 4.2 | 1.68 | 0.02 | 2.0 | 3.8 | 94% | 46.1 | [1] |
| *Pyrococcus horikoshi shinkaj* OT3**^4^** | 172 | 170 | 9 | ND | ND | ND | 1.0 | 1.1 | 7% | 8.3 | [2] |
| *Caenorhabditis elegans* | 2,847 | 4,584 | 13 | 4.8 | 1.62 | 0.02 | 1.6 | 3.1 | 83% | 12.5 | [3] |
| *C. elegans* (High Confidence) | 1,415 | 2,157 | 13 | 4.9 | 1.61 | 0.03 | 1.5 | 2.9 | 79% | 6.2 | [3] |
| *Plasmodium falciparum* | 1,308 | 2,846 | 10 | 4.3 | 1.8 | 0.03 | 2.2 | 4.2 | 93% | 24.7 | [4] |
| *Drosophila* (High confidence) | 4,591 | 4,972 | 27 | 9.4 | 2.7 | 0.04 | 1.1 | 2 | 42% | 32.8 | [5] |
| *Drosophila* (All) | 7,488 | 24,408 | 11 | 4.3 | 1.95 | 0.02 | 3.3 | 6 | 96% | 53.6 | [6] |
| *Escherichia coli* K12 | 3,427 | 17,257 | 9 | 3.3 | 1.4 | 0.11 | 5.0 | 9.6 | 98% | 80.2 | [7, 8] |
| Human (All) | 2,962 | 5,902 | 11 | 4.8 | 1.8 | 0.01 | 2.0 | 3.9 | 85% | 12.3 | [9, 10] |

**^1^**Network statistics were compiled using Cytoscape v2.1 [11] and the Plug-in program Network Analyzer [12].

**^2^**Connected Pairs refers to the percentage of the interactions within the single largest connected component.

**^3^**CampyYTH v3.1 dataset.

**^4^**Network features were not determined since only 7% (12 total) of the interactions were contained within a single network.

**References:**

1. Rain JC, Selig L, De Reuse H, Battaglia V, Reverdy C, Simon S, Lenzen G, Petel F, Wojcik J, Schachter V *et al*: **The protein-protein interaction map of *Helicobacter pylori***. *Nature* 2001, **409**(6817):211-215.

2. Usui K, Katayama S, Kanamori-Katayama M, Ogawa C, Kai C, Okada M, Kawai J, Arakawa T, Carninci P, Itoh M *et al*: **Protein-protein interactions of the hyperthermophilic archaeon *Pyrococcus horikoshii* OT3**. *Genome Biol* 2005, **6**(12):R98.

3. Li S, Armstrong CM, Bertin N, Ge H, Milstein S, Boxem M, Vidalain PO, Han JD, Chesneau A, Hao T *et al*: **A map of the interactome network of the metazoan *C. elegans***. *Science* 2004, **303**(5657):540-543.

4. LaCount DJ, Vignali M, Chettier R, Phansalkar A, Bell R, Hesselberth JR, Schoenfeld LW, Ota I, Sahasrabudhe S, Kurschner C *et al*: **A protein interaction network of the malaria parasite *Plasmodium falciparum***. *Nature* 2005, **438**(7064):103-107.

5. Giot L, Bader JS, Brouwer C, Chaudhuri A, Kuang B, Li Y, Hao YL, Ooi CE, Godwin B, Vitols E *et al*: **A protein interaction map of *Drosophila melanogaster***. *Science* 2003, **302**(5651):1727-1736.

6. Pacifico S, Liu G, Guest S, Parrish JR, Fotouhi F, Finley RL, Jr.: **A database and tool, IM Browser, for exploring and integrating emerging gene and protein interaction data for *Drosophila***. *BMC Bioinformatics* 2006, **7**(1):195.

7. Butland G, Peregrin-Alvarez JM, Li J, Yang W, Yang X, Canadien V, Starostine A, Richards D, Beattie B, Krogan N *et al*: **Interaction network containing conserved and essential protein complexes in *Escherichia coli***. *Nature* 2005, **433**(7025):531-537.

8. Arifuzzaman M, Maeda M, Itoh A, Nishikata K, Takita C, Saito R, Ara T, Nakahigashi K, Huang HC, Hirai A *et al*: **Large-scale identification of protein-protein interaction of *Escherichia coli* K-12**. *Genome Res* 2006, **16**(5):686-691.

9. Rual JF, Venkatesan K, Hao T, Hirozane-Kishikawa T, Dricot A, Li N, Berriz GF, Gibbons FD, Dreze M, Ayivi-Guedehoussou N *et al*: **Towards a proteome-scale map of the human protein-protein interaction network**. *Nature* 2005, **437**(7062):1173-1178.

10. Stelzl U, Worm U, Lalowski M, Haenig C, Brembeck FH, Goehler H, Stroedicke M, Zenkner M, Schoenherr A, Koeppen S *et al*: **A human protein-protein interaction network: a resource for annotating the proteome**. *Cell* 2005, **122**(6):957-968.

11. Shannon P, Markiel A, Ozier O, Baliga NS, Wang JT, Ramage D, Amin N, Schwikowski B, Ideker T: **Cytoscape: a software environment for integrated models of biomolecular interaction networks**. *Genome Res* 2003, **13**(11):2498-2504.

12. www.cytoscape.org/plugins2.php
