# Supplementary material for: A proteome-wide protein interaction map for Campylobacter jejuni
Source: Genome Biol. 2007 Jul 5;8(7):R130. doi: 10.1186/gb-2007-8-7-r130 (PMC2323224; doi:10.1186/gb-2007-8-7-r130)
Supplement: Additional data file 6 — Conserved subnetworks between C. jejuni and E. coli or C. jejuni and yeast [file gb-2007-8-7-r130-S6.doc]

| **Additional Data File 6.** Conserved subnetworks between *C. jejuni* and *E. coli* and *C. jejuni* and yeast. | | | | | | |
| --- | --- | --- | --- | --- | --- | --- |
|  |  |  | **Organism Compared with *C. jejuni*** | **Gene ontology categories enriched in the conserved subnetworks** | | |
| **Subnetwork ID** | **P Value^1^** | **P Value^2^** |  | **Biological Process** | **Molecular Function** | **Cellular Component** |
| 0 | 0.000086 | 0.00E+00 | *E. coli* | protein metabolism | purine nucleotide binding | None |
| 2 | 0.000315 | 3.38E-03 | *E. coli* | metabolism | transferase activity | None |
| 33 | 0.00164 | 1.01E-02 | *E. coli* | cellular macromolecule metabolism | purine nucleotide binding | None |
| 37 | 0.001803 | 1.01E-02 | *E. coli* | protein metabolism | None | cytoplasm |
| 52 | 0.002262 | 1.01E-02 | *E. coli* | cellular macromolecule metabolism | transferase activity | None |
| 69 | 0.003977 | 1.35E-02 | *E. coli* | macromolecule metabolism | protein binding | None |
| 70 | 0.003991 | 1.35E-02 | *E. coli* | metabolism | None | None |
| 72 | 0.004186 | 1.35E-02 | *E. coli* | protein metabolism | None | cytoplasm |
| 79 | 0.004442 | 1.35E-02 | *E. coli* | cellular macromolecule metabolism | None | None |
| 84 | 0.004574 | 1.35E-02 | *E. coli* | protein metabolism | purine nucleotide binding | None |
| 96 | 0.005676 | 1.35E-02 | *E. coli* | protein metabolism | nucleic acid binding | None |
| 100 | 0.006 | 2.03E-02 | *E. coli* | cellular macromolecule metabolism | protein binding | None |
| 126 | 0.006422 | 2.03E-02 | *E. coli* | cellular macromolecule metabolism | ion binding | None |
| 129 | 0.007004 | 2.70E-02 | *E. coli* | cellular biosynthesis | protein binding | None |
| 142 | 0.009026 | 2.70E-02 | *E. coli* | protein metabolism | nucleic acid binding | None |
| 155 | 0.010953 | 2.70E-02 | *E. coli* | cellular macromolecule metabolism | transferase activity | None |
| 160 | 0.013047 | 4.05E-02 | *E. coli* | protein metabolism | None | cytoplasm |
| 163 | 0.013545 | 4.05E-02 | *E. coli* | None | None | None |
| 170 | 0.014453 | 4.39E-02 | *E. coli* | cellular macromolecule metabolism | ion binding | None |
| 178 | 0.015565 | 4.39E-02 | *E. coli* | None | purine nucleotide binding | None |
| 183 | 0.015831 | 4.73E-02 | *E. coli* | cellular biosynthesis | protein binding | None |
| 184 | 0.015855 | 4.73E-02 | *E. coli* | protein metabolism | nucleic acid binding | None |
| 188 | 0.015996 | 4.73E-02 | *E. coli* | protein metabolism | None | None |
| 236 | 0.023084 | 5.07E-02 | *E. coli* | cellular macromolecule metabolism | purine nucleotide binding | None |
| 240 | 0.023429 | 5.41E-02 | *E. coli* | biopolymer metabolism | nucleic acid binding | None |
| 250 | 0.025917 | 5.41E-02 | *E. coli* | protein metabolism | nucleic acid binding | None |
| 260 | 0.027226 | 5.41E-02 | *E. coli* | cellular macromolecule metabolism | None | None |
| 262 | 0.029123 | 5.41E-02 | *E. coli* | cellular macromolecule metabolism | protein binding | None |
| 276 | 0.031008 | 5.41E-02 | *E. coli* | cellular macromolecule metabolism | protein binding | None |
| 279 | 0.032152 | 5.41E-02 | *E. coli* | protein metabolism | nucleic acid binding | None |
| 287 | 0.032668 | 5.41E-02 | *E. coli* | None | None | None |
| 288 | 0.032708 | 5.41E-02 | *E. coli* | None | None | None |
| 289 | 0.032755 | 5.74E-02 | *E. coli* | protein metabolism | nucleic acid binding | None |
| 290 | 0.033043 | 5.74E-02 | *E. coli* | metabolism | None | None |
| 300 | 0.03496 | 6.08E-02 | *E. coli* | protein metabolism | purine nucleotide binding | None |
| 307 | 0.036848 | 6.08E-02 | *E. coli* | metabolism | None | None |
| 311 | 0.037191 | 6.42E-02 | *E. coli* | protein metabolism | nucleic acid binding | None |
| 314 | 0.037739 | 6.42E-02 | *E. coli* | cellular macromolecule metabolism | protein binding | None |
| 321 | 0.039344 | 6.42E-02 | *E. coli* | protein metabolism | nucleic acid binding | None |
| 324 | 0.039804 | 6.42E-02 | *E. coli* | cellular macromolecule metabolism | nucleic acid binding | None |
| 330 | 0.041172 | 6.42E-02 | *E. coli* | protein metabolism | None | cytoplasm |
| 341 | 0.045292 | 6.76E-02 | *E. coli* | protein metabolism | None | None |
| 344 | 0.046422 | 6.76E-02 | *E. coli* | protein metabolism | None | None |
| 345 | 0.047677 | 7.43E-02 | *E. coli* | protein metabolism | purine nucleotide binding | None |
| 346 | 0.047873 | 7.43E-02 | *E. coli* | metabolism | purine nucleotide binding | None |
| 349 | 0.048179 | 7.43E-02 | *E. coli* | protein metabolism | nucleic acid binding | cytoplasm |
| 351 | 0.048311 | 7.43E-02 | *E. coli* | protein metabolism | None | cytoplasm |
| 353 | 0.048659 | 7.43E-02 | *E. coli* | cellular macromolecule metabolism | protein binding | None |
| 0 | 0 | 0.00E+00 | *S. cerevisiae* | metabolism | None | cytoplasm |
| 15 | 0.00053 | 0.00E+00 | *S. cerevisiae* | metabolism | None | None |
| 18 | 0.0006 | 0.00E+00 | *S. cerevisiae* | metabolism | None | None |
| 20 | 0.00093 | 0.00E+00 | *S. cerevisiae* | metabolism | None | None |
| 40 | 0.00228 | 5.00E-02 | *S. cerevisiae* | response to stimulus | None | None |
| 44 | 0.00273 | 5.00E-02 | *S. cerevisiae* | metabolism | None | intracellular non-membrane-bound organelle |
| 56 | 0.00444 | 7.50E-02 | *S. cerevisiae* | metabolism | None | None |
| 57 | 0.00469 | 7.50E-02 | *S. cerevisiae* | metabolism | None | None |
| 61 | 0.0051 | 7.50E-02 | *S. cerevisiae* | response to stimulus | None | intracellular non-membrane-bound organelle |
| 62 | 0.00541 | 8.75E-02 | *S. cerevisiae* | metabolism | None | None |
| 66 | 0.00606 | 1.13E-01 | *S. cerevisiae* | metabolism | None | None |
| 81 | 0.0077 | 1.38E-01 | *S. cerevisiae* | metabolism | None | None |
| 84 | 0.01655 | 1.63E-01 | *S. cerevisiae* | metabolism | None | None |
| 88 | 0.01827 | 1.63E-01 | *S. cerevisiae* | metabolism | None | None |
| 102 | 0.02433 | 1.88E-01 | *S. cerevisiae* | metabolism | None | None |
| 106 | 0.03274 | 2.00E-01 | *S. cerevisiae* | metabolism | None | cytoplasm |
| 113 | 0.04377 | 2.38E-01 | *S. cerevisiae* | metabolism | None | None |
| 117 | 0.04578 | 2.50E-01 | *S. cerevisiae* | biosynthesis | None | None |
| 121 | 0.04955 | 2.63E-01 | *S. cerevisiae* | organic acid metabolism | None | cytoplasm |

^1^Calculated based on 1000 random networks in which the degree of each protein was maintained (see materials and methods).

^2^Calculated based on 1000 random networks for which the topology matched the *C. jejuni* interaction map (see materials and methods).
