# Supplementary figures and images for: A proteome-wide protein interaction map for Campylobacter jejuni
Source: Genome Biol. 2007 Jul 5;8(7):R130. doi: 10.1186/gb-2007-8-7-r130 (PMC2323224; doi:10.1186/gb-2007-8-7-r130)

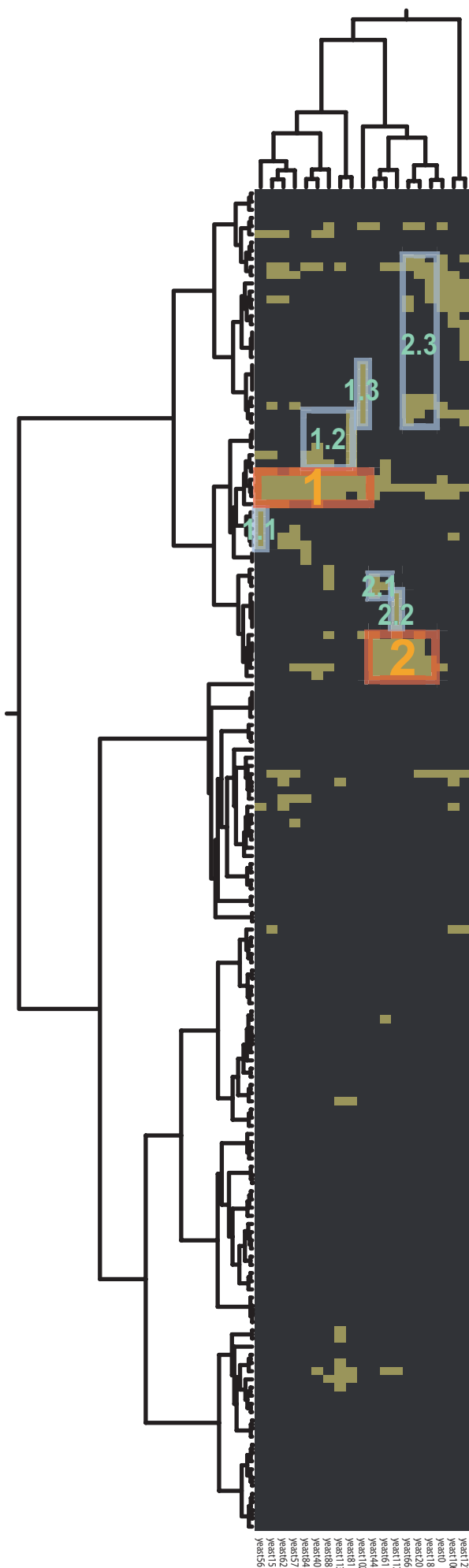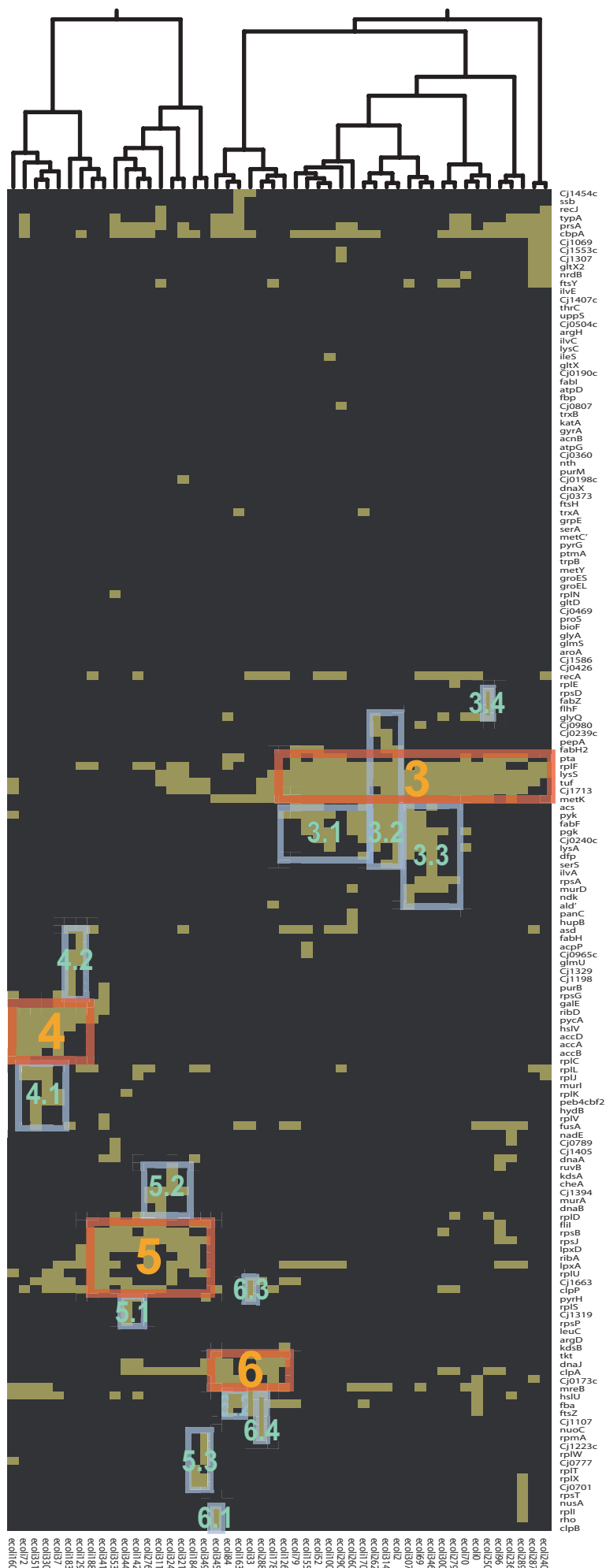

Supplement: Additional data file 8 — Higher resolution version of Figure 5, showing hierarchical clustering of conserved subnetworks [file gb-2007-8-7-r130-S8.pdf]
