# Supplementary material for: A proteome-wide protein interaction map for Campylobacter jejuni
Source: Genome Biol. 2007 Jul 5;8(7):R130. doi: 10.1186/gb-2007-8-7-r130 (PMC2323224; doi:10.1186/gb-2007-8-7-r130)
Supplement: Additional data file 9 — Enriched functions within the cores and modules of Figure 5 [file gb-2007-8-7-r130-S9.doc]

**Additional Data File 9.** Enriched functions within cores and modules^1^

| **Cores and Modules** | **Enriched functions** |
| --- | --- |
| **Core 1** | **Serine family amino acid metabolism** |
| Module 1-1 | Serine family amino acid biosynthesis |
| Module 1-2 | Generation of precursor metabolites and energy |
| Module 1-3 | Oxygen and reactive oxygen species |
| **Core 2** | **One carbon compound metabolism** |
| Module 2-1 | Protein folding |
| Module 2-2 | Nitrogen compound metabolism |
| Module 2-3 | Oxygen and reactive oxygen species |
| **Core 3** | **Lysyl-tRNA aminoacylation** |
| Module 3-1 | Acetate metabolism |
| Module 3-2 | Macromolecule metabolism |
| Module 3-3 | Metabolism |
| **Core 4** | **Organic acid biosynthesis** |
| Module 4-1 | Fatty acid metabolism |
| Module 4-2 | Carboxylic acid biosynthesis |
| **Core 5** | **Macromolecule metabolism** |
| Module 5-1 | Macromolecule metabolism (nucleotide-sugar metabolism) |
| Module 5-2 | Macrometabolism (cellular macrometabolism) |
| Module 5-3 | Macrometabolism (protein metabolism) |
| **Core 6** | **Protein folding** |
| Module 6-1 | Response to biotic stimulus |
| Module 6-2 | Macromolecule metabolism (protein polymerization) |
| Module 6-3 | Macromolecule metabolism (lipid A metabolism) |
| Module 6-4 | Macromolecule metabolism |

^1^See Additional data file 8 for Core and Module protein and subnetwork membership.
