# Supplementary material for: A proteome-wide protein interaction map for Campylobacter jejuni
Source: Genome Biol. 2007 Jul 5;8(7):R130. doi: 10.1186/gb-2007-8-7-r130 (PMC2323224; doi:10.1186/gb-2007-8-7-r130)
Supplement: Additional data file 11 — Essential proteins interact with each other more often than expected by chance [file gb-2007-8-7-r130-S11.doc]

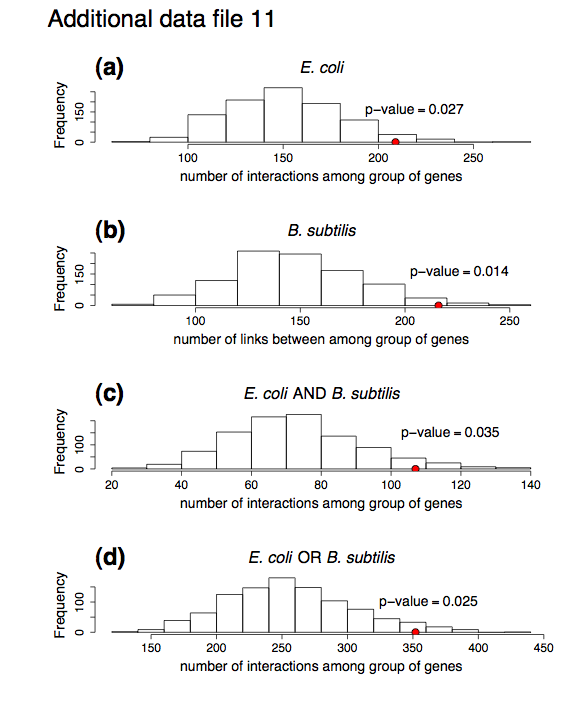


**Additional data file 11.** **Essential proteins interact with each other more often than expected by chance.** The red circles correspond to the numbers of interactions among the indicated set of essential proteins. Essential protein sets were generated as described in Materials and Methods. We selected 1000 random groups (of the same size as the essential set) of proteins and counted the number of interactions among them (black-lined rectangles). These results were generated from CampyYTH v3.1 using all of the data. This relationship was not observed for high confidence data only.
