# Supplementary material for: A proteome-wide protein interaction map for Campylobacter jejuni
Source: Genome Biol. 2007 Jul 5;8(7):R130. doi: 10.1186/gb-2007-8-7-r130 (PMC2323224; doi:10.1186/gb-2007-8-7-r130)
Supplement: Additional data file 14 — Supplementary materials and methods [file gb-2007-8-7-r130-S14.doc]

**Additional Data File 14. Supplemental Methods**

**Assignment of confidence scores**

Confidence scores were determined for each interaction based on methods described by Bader et al. [1, 2]. We fit a generalized linear model [3] using experimental and topological attributes of yeast two-hybrid interactions, including the number of interactions for each protein in a pair, Leu2 and *lacZ* reporter activity, number of times detected, number of orientations detected (BD-AD and AD-BD), and the Jaccard [4] local clustering value (for an interaction between A and B, Jaccard is the number of proteins interacting with both A and B divided by the number of proteins interacting with either A or B, excluding A and B themselves). Fitting the model required both positive and negative training sets. Because a reference set of known interactions is not available for *C. jejuni*, we derived a set of positive training data (85 interactions total) by assuming that the conserved interactions (reciprocal best match interologs) in common with either the *E. coli* low-throughput interaction set [5], the *H. pylori* yeast two-hybrid set [6], or the *E. coli* protein complex set [7] are likely to be true positives. We derived a set of likely true negatives (111 total) for the negative training data by considering interactions between proteins whose orthologs in *E. coli* or *H. pylori* were separated in the respective interaction maps by greater than the average distance of all pairs (>= 4). Positive and negative training cases were weighted inversely to the number of interactions in each set. When training sets are weighted this way, a confidence score greater than 0.5 means that available data and features support that a specific interaction has a better than random chance to be a true interaction, so that it allows 0.5 to be used as the threshold between high and low confidence interactions. Validation using protein features not used in the scoring system support the choice of 0.5 as a threshold for higher confidence interactions (discussed below and see Figure 2c). The glm function in the R environment [8] was used to fit our logistic model. We adopted a stepwise procedure to find the best possible model based on our training data. Single attribute terms were added to the model one by one; interactions between any two terms were then added to the model. Of the attributes tested, the numbers of interactions per protein were found to be negative predictors of biologically relevant interactions, while reporter activities were positive predictors. The local clustering property, Jaccard was also found to be a positive predictor, but its effect was masked by the reporter activity attributes so it was not included in the final model. Two other features correlated with true positives but were not included in the final scoring model. Interactions detected in both orientations (BD-AD and AD-BD) were more likely to be in the true positive training set, but only 141 interactions had this property making it a weak predictor for the entire data set. Likewise, the number of times an interaction was detected in the library screening approach correlated with likelihood of being in the true positive training set, but only 132 interactions were detected using this approach.

To evaluate the scoring model, we performed a stratified five-fold cross validation. We split our training data into five approximately equal sets, each containing about the same number of positives and negatives. We then used four sets to train a logistic model, and evaluated the training model using the fifth unused set. This process was repeated five times so that each data point was used at least once for both training and testing. We then computed how accurate the model was and how many positives could be successfully recaptured. Cross validation reported a precision of 91.4% and a recall of 78.9%, which gave us confidence that it is a reasonably well-fitted model. We then used the full sets of positives and negatives in training and obtained our final logistic model. The final model was used to compute confidence scores for 15,949 initial positive interactions prior to retesting. Of these, 3,209 scored higher than 0.5, which we define as the High Confidence Set (HCS). Of the interactions with high confidence scores (>0.5), 90% corresponded to interactions that had repeated when retested, while only 68% of the low confidence interactions had repeated. The final confidence model is shown below.

Confidence Model v1.0

glm(formula = positive ~ log(Gene1_interactions_as_BD) + log(Gene1_interactions_as_AD) + log(Gene2_interactions_as_AD) + log(Gene2_interactions_as_BD) + sqrt(c_lacZ) + sqrt(c_Sum), family = binomial, data = newtraining, y = TRUE)

Deviance Residuals:

| Min | 1Q | Median | 3Q | Max |
| --- | --- | --- | --- | --- |
| -2.4783 | -0.539 | -0.3777 | 0.2489 | 2.3872 |

Coefficients:

|  | Estimate | Std. Error | z value | Pr(>|z|) |
| --- | --- | --- | --- | --- |
| (Intercept) | -2.625310 | 0.583243 | -4.501 | 6.76e-06 |
| log(Gene1_interactions_as_BD) | -0.453496 | 0.062703 | -7.232 | 4.74e-13 |
| log(Gene1_interactions_as_AD) | -0.132831 | 0.021602 | -6.149 | 7.80e-10 |
| log(Gene2_interactions_as_AD) | -0.258112 | 0.078393 | -3.293 | 0.000993 |
| log(Gene2_interactions_as_BD) | 0.006979 | 0.008660 | 0.806 | 0.420292 |
| sqrt(c_lacZ) | 1.704084 | 0.175202 | 9.726 | < 2e-16 |
| sqrt(c_Sum) | 3.016407 | 0.314952 | 9.577 | < 2e-16 |

The following equation, derived from the model mentioned above, was used to compute the final confidence scores for all interactions.

x = -0.453496*log(Gene1_interactions_as_BD) - 0.132831*log(Gene1_interactions_as_AD) - 0.258112*log(Gene2_interactions_as_AD) + 0.006979*log(Gene2_interactions_as_BD) + 1.704084*sqrt(c_lacZ) + 3.016407*sqrt(c_Sum)-2.625310

p = exp(x)/(1+exp(x));

where p is the final confidence score for an interaction.

**Comparative network analysis**

Protein-protein interactions from *C. jejuni* were compared with those from *E. coli* [7]; *H. pylori* [6]; and *S. cerevisiae* from the Database of Interacting Proteins [5]. Corresponding protein sequences were obtained from the following sources: *C. jejuni* NCTC11168 [9]; *E. coli* [10]; *H. pylori* [11]; and *S. cerevisiae* [12]. We used NetworkBlast to identify significant conserved protein-protein interaction subnetworks [13]. A stand-alone Java version of the program is available at [14]. Briefly, the algorithm takes as input a pair of protein-protein interaction networks (and optionally, associated probabilities of interactions), one for each of two species, along with a set of homology relationships between the proteins of the two networks. We constructed the homology relationships from an all-versus-all BLAST of the complete set of protein sequences for each of the two species, taking the top 10 hits with E-value <= 10^-10^. Next, a network alignment graph was created where each node represents a homologous pair of proteins from species 1 and 2 (e.g., a1 and a2) and each edge represents a conserved interaction (a1/a2 connects to b1/b2 if the a-b interaction is found in both species; interactions may be either direct (distance 1) or indirect (distance 2), in which a-b is connected through a common neighbor, i.e., a-c-b). A greedy search is initiated from each node to identify conserved protein subnetworks, defined as dense subgraphs within the network alignment graph (of maximum size 15 proteins per species). When multiple subnetworks contain protein homologs that overlap by >=50%, only the complex with the highest density was included in the final result. The significance of the conserved subnetworks was evaluated using permutation testing, in which NetworkBlast was run on 1000 protein interaction networks where the interactions were randomized holding both the degree of each protein and its homology the same. This process generated a background distribution of complex scores (interaction densities); a significance threshold was chosen at the top five percent of this distribution. From this background distribution we calculated two sets of *p-*values: the first assuming the random distribution described above reflected all possible scores and the second by generating another random distribution created by randomizing the labels of the input interaction network, thereby keeping the network topology constant. To further assess the significance of the identified complexes, we also calculated their Benjamini False Discovery Rates (FDRs) defined as the percentage of expected false positives in the returned set [15]. Here, we calculated FDR as the average number of random subnetworks above the threshold divided by the number of real scores above the threshold. The FDR for the conserved subnetworks from *E. coli* equaled 0.297, and for the *S. cereviseae* conserved subnetworks, the FDR equaled 0.098. GO annotations [16] of proteins in each conserved complex were analyzed to identify significant functional enrichments (Additional data file 6). We calculated a hypergeometric *p*-value of enrichment for each GO annotation in the three divisions of the GO hierarchy and constrained the annotations by requiring that at least half of the proteins in a complex ascribe to the enrichment. The most specific annotations with hypergeometric *p*-value < 0.05 in each of the three divisions were then assigned to each complex. A complete list of conserved complexes between *C. jejuni* and *E. coli* or *S. cerevisiae* is available for download at [17]. The significant conserved subnetworks provided predictions of 379 new *C. jejuni* protein-protein interactions not found in the two-hybrid screens (Additional data file 7). A protein pair (a,b) was predicted to interact directly if: 1., both a and b were present in the same significant conserved complex; 2., this pair was observed to interact indirectly in *C. jejuni*; and 3., this pair corresponded to a direct interaction in the comparison species’ network.

**Clustering of conserved subnetworks**

Since proteins can belong to more than one complex, we clustered the significant conserved subnetworks by protein membership, in effect ‘superclustering’ the interactions (Figure 5b). An *n*×*m* matrix was constructed, where *n* is the number of significant subnetworks and *m* is the number of unique proteins involved in any of the significant subnetworks. Using the open source tool ClustArray [18], we clustered the proteins hierarchically with UPGMA and clustered the subnetworks with a combination *k*-means algorithm followed by UPGMA hierarchical clustering. The number of clusters *k=3* was chosen as the parameter that approximately minimized within-cluster variability and maximized between-cluster variability (data not shown). Identities of complexes and proteins are shown in the high resolution version of the hierarchical clustering in Additional data file 8. Lists of the proteins comprising complexes are available for download at [17].

**References**
